# Molecular Characterization and Co-expression Analysis of the *SnRK2* Gene Family in Sugarcane (*Saccharum officinarum* L.)

**DOI:** 10.1038/s41598-017-16152-4

**Published:** 2017-12-15

**Authors:** Changning Li, Qian Nong, Jinlan Xie, Zeping Wang, Qiang Liang, Manoj Kumar Solanki, Mukesh Kumar Malviya, Xiaoyan Liu, Yijie Li, Reemon Htun, Jiguang Wei, Yangrui Li

**Affiliations:** 1 College of Agriculture, State Key Laboratory of Conservation and Utilization of Subtropical Agro-bioresources, Guangxi University, Nanning, Guangxi 530004 China; 2 Key Laboratory of Sugarcane Biotechnology and Genetic Improvement (Guangxi), Ministry of Agriculture, Guangxi Key Laboratory of Sugarcane Genetic Improvement, Sugarcane Research Center of Chinese Academy of Agricultural Sciences, Nanning, Guangxi 530007 China

## Abstract

In plants, both abscisic acid (ABA) dependent and independent pathways form the basis for the response to environmental stresses. Sucrose non-fermenting 1-related protein kinase 2 (SnRK2) plays a central role in plant stress signal transduction. However, complete annotation and specific expression patterns of *SnRK2s* in sugarcane remain unclear. For the present study, we performed a full-length cDNA library survey of sugarcane, thus identifying ten *SoSnRK2* genes via phylogenetic, local BLAST methods, and various bioinformatics analyses. Phylogenetic analysis indicated division of *SoSnRK2* genes into three subgroups, similar to other plant species. Gene structure comparison with *Arabidopsis* suggested a unique evolutionary imprint of the *SnRK2* gene family in sugarcane. Both sequence alignment and structural annotation provided an overview of the conserved N-terminal and variations of the C-terminal, suggesting functional divergence. Transcript and transient expression assays revealed *SoSnRK2s* to be involved in the responses to diverse stress signals, and strong ABA induction of *SoSnRK2s* in subgroup III. Co-expression network analyses indicated the existence of both conserved and variable biological functions among different *SoSnRK2s* members. In summary, this comprehensive analysis will facilitate further studies of the *SoSnRK2* family and provide useful information for the functional validation of *SoSnRK2s*.

## Introduction

In their natural habitat, plants are repeatedly exposed to environmental stresses such as drought, high salinity, extreme temperatures, and pathogen infection, affecting both biomass and crop yield. While dealing with these stresses to ensure survival and completion of their life cycles, plants have developed various defense mechanisms^1,2^, of which stress signals recognized and transmitted by specialized signaling pathways are involved in such mechanism. Protein kinases and phosphatases are considered as central components of these pathways^3^. Protein kinases involved in stress signaling are common throughout all eukaryotic organisms and most specifically in plants containing calcium-dependent protein kinases (CDPKs), Glycogen synthase kinase 3 (GSK3), mitogen-activated protein kinases (MAPKs), and sucrose non-fermenting 1 (SNF1) -related protein kinases (SnRKs)^4–6^. Plant SnRKs can be divided into SnRK1, SnRK2, and SnRK3 based on characteristics of sequence similarity, domain structure, and cellular functions^7^. Among these subfamilies, SnRK2s are unique. Accumulated evidence shows that SnRK2s are playing an important role in ABA-mediated signaling pathways and plant response to abiotic stresses, especially osmotic stress^8,9^. Furthermore, some SnRK2s have an important function in the regulation of seed dormancy, germination, maturation, seedling growth, and flowering time, as well as stomata movements during drought stress^10–13^. Analyses of amino acid sequence indicated that all SnRK2s have: i) a conserved N-terminal catalytic domain, similar to SNF1/AMP kinases; this is a requirement for kinase activity, and ii) a variable adjustable domain at the C-terminal required for the physical interaction between SnRK2s and type 2 C protein phosphatases (PP2Cs). The latter are key components in the ABA signaling pathway^9,11^.

Before the year 2000, only limited knowledge was available of how ABA and abiotic stresses induce *SnRK2* genes^14,15^, despite earlier studies recognizing the role of SnRK2s as enzymes involved in stress signaling of plants^16^. To date, the *SnRK2s* have been identified in the genomes of numerous plants, including rice^17^, maize^18^, sorghum^19^, apple^20^, pakchoi^21^, grape^22^, and wheat^23^, and a significant amount of research has focused on the involvement of *SnRK2s* in stress signaling pathways. In *Arabidopsis*, ten *SnRK2s* have been identified and named *AtSnRK2*.*1* to *AtSnRK2*.*10* ^24^; all of these *AtSnRK2s* except for *AtSnRK2*.*9* are stimulated by hyperosmotic and saline stresses, and five of the remaining nine *AtSnRK2s* being activated by ABA^7,24^. Among those, *AtSnRK2*.*2/2*.*3/2*.*6* are central to the ABA signal transduction network and act as the main positive regulators of ABA signaling in response to environmental stress^9,25^. All *SnRK2s* in *Oryza sativa* (named *OsSAPK1- OsSAPK10*) are activated by hyperosmotic stress, and three (*OsSAPK8/9/10*) are additionally activated by ABA^26^. *OsSAPK4* plays a role in the salt stress response by regulating genes with ion homeostatic and oxidative stress response functionality^27^. Overexpression of *AtSnRK2*.*8* and *OsSAPK4* increased drought and salt tolerance in transgenic plants significantly^27,28^. Overexpression of *AtSnRK2*.*6* in *Arabidopsis* increased sucrose and fatty acid metabolism in the leaves, and the metabolic alterations were accompanied by amelioration of those physiological processes that require high levels of carbon and energy input^29^. Overexpression of the wheat *SnRK2* genes *TaSnRK2*.*4*, *TaSnRK2*.*7*, and *TaSnRK2*.*8* in *Arabidopsis* enhanced tolerance to multi-abiotic stresses^30–32^. The soybean gene *GsAPK* can be activated via drought, salinity, cold, and ABA stress, and its overexpression in *Arabidopsis* altered the plant tolerance to high salinity and ABA stress^33^. Overexpression of *SoSnRK2*.*1* (named *SoSnRK*2.3 in our study) of sugarcane enhanced drought tolerance in tobacco^34^. In summary, solid evidence indicates that the *SnRK2* gene family is involved in multi-environmental stress responses and all genes of this family have potential use in the improvement of abiotic stress tolerance and yield enhancement^35^.

Sugarcane is a crop of great economic importance, contributing to approximately 75% of the global sugar production; furthermore, it is increasingly becoming relevant for the production of renewable energy. Since it grows in tropical and sub-tropical areas, sugarcane is vulnerable to climatic changes, especially to abiotic stresses such as water stress and low temperature. Although extensive progress has been made in abiotic stress research, the *SnRK2s* research in sugarcane remains very limited. In this article, ten *SnRK2s* have been cloned based on the sequence similarity between sugarcane and other plant species. Furthermore, these have been identified via multiple bioinformatic analyses including gene classification, sequence alignment, gene structure, and phylogenetic analyses. Moreover, the expression patterns of *SnRK2s* in different sugarcane tissues, under various growth stages, and in co-expression networks responding to abiotic stresses and ABA treatment were analyzed via quantitative real time-PCR (qRT-PCR) and a transient expression assay. These systematical analyses will provide a foundation for further functional studies to reveal the biological function of *SnRK2s* in sugarcane.

## Results

### *SoSnRK2*s identification in sugarcane

**Figure 1:**
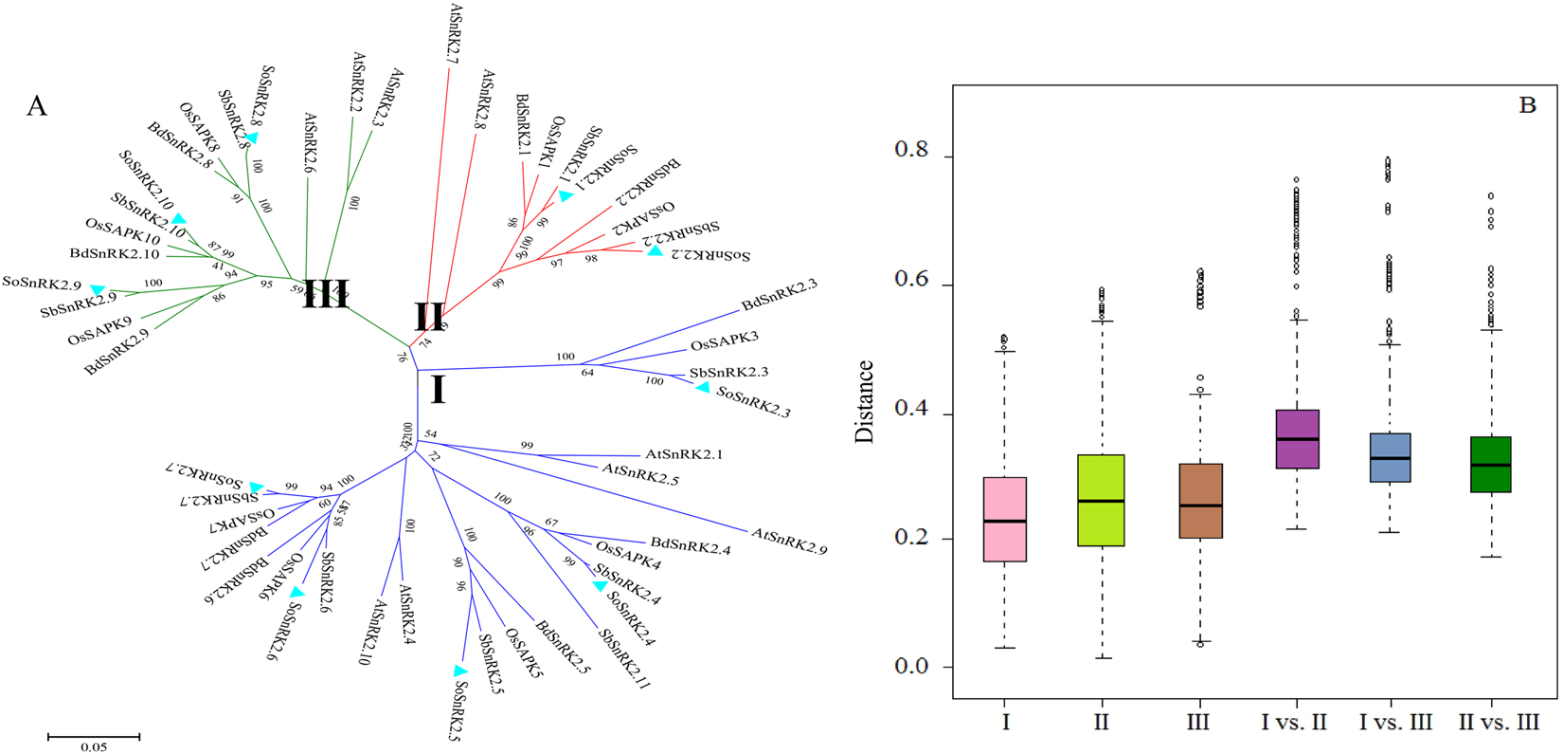
Phylogenetic relationships among *SnRK2* genes in five plant species (**A**) and genetic distances among different groups of *SnRK2* genes (**B**). Group I, II, and III are shaded blue, red, and green, respectively. Short species names in (A) are *A*. *thaliana* (*At*), *S*. *officinarum* (*So*), *O*. *sative* (*Os*), *B*. *distachyon* (*Bd*), and *S*. *bicolor* (*Sb*), respectively. The phylogenetic tree was constructed using the maximum likelihood (ML) method and bootstrap values were calculated with 1000 replications via MEGA7.0 software. The *SnRK2s* of *S*. *officinarum* are indicated with cyan triangles.

Ten *SoSnRK2* genes (*SoSnRK2*.*1-2*.*10*) were obtained by screening full-length cDNA libraries. The ORF of *SoSnRK2s* ranged from 1002 to 1227 bp, encoding polypeptides of 333 to 408 aa. The predicted molecular mass and pI were ranged from 37.8 to 45.4 kDa and 4.80 to 6.51, respectively (Supplementary Table S1). To assess the relationships amongst the *SnRK2s* in different plants, a phylogenetic tree was constructed with the deduced amino acid sequences. Based on this phylogenetic analysis, ten *SoSnRK2* genes were divided into three different groups, of which *SoSnRK2*.*1*-*2*.*2* belong to group II, *SoSnRK2*.*3*-*2*.*7* belongs to group I, and *SoSnRK2*.*8*-*2*.*10* were assigned to group III. The deduced amino acid sequence showed high homology with counterpart monocot *SnRK2* members, based on the clustering of these species in the phylogenetic analysis (Fig. 1A). The genetic distances among the three *SoSnRK2* groups were analyzed (Fig. 1B), of which Group II and group III were most closely related, while group I and group II were the least closely related. Given previous findings, indicating that group III is the most ancient and group I is the most recent group^36^, we inferred that both group I and group II originated from group III.

### SoSnRK2s sequence analysis

**Figure 2:**
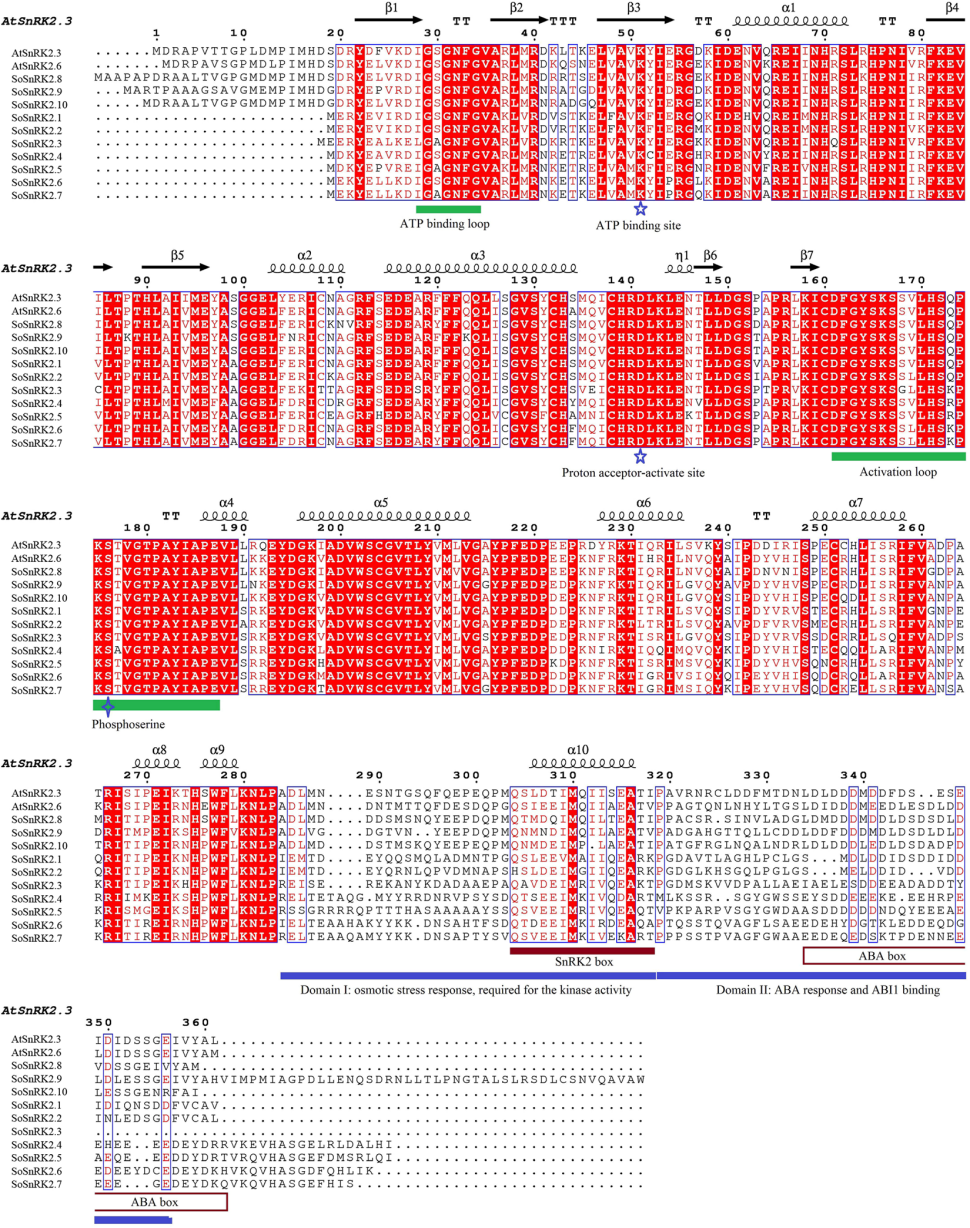
Structure-based sequence alignment of the SoSnRK2s with AtSnRK2.3 and AtSnRK2.6. Red background shows sequence identity and red letters show sequence similarity in the alignment. Secondary structure elements and ATP binding loop, activation loop, proton acceptor active site, SnRK2 box, and ABA box are indicated. The alignment was performed via BioEdit, with the program ClustalW and the similarity matrix BLOSUM62 with default parameters.

Secondary structure prediction revealed that SoSnRK2s formed ten α-helixes and seven β-pleated sheets (Fig. 2). Sequence alignment indicated that SoSnRK2s have the potential for serine/threonine and tyrosine kinase activities. Similar to other SnRK2s, SoSnRK2s has two domains in its N- and C-terminal regions. The N-terminal catalytic domain is highly conserved which containing an ATP-binding site and an ATP-binding loop. The aspartic acid, serine (represent for proton acceptor activate site) and phosphoserine were highly conserved in all SoSnRK2s. In addition to the conserved kinase domain, SoSnRK2s contain a C-terminal regulatory region that encompasses two conserved motifs: the SnRK2 box, which is required for kinase activity via an unknown mechanism, and the highly acidic ABA box, which is important to mediate SnRK2s interactions with type 2 C protein phosphatases (Fig. 2).

**Figure 3:**
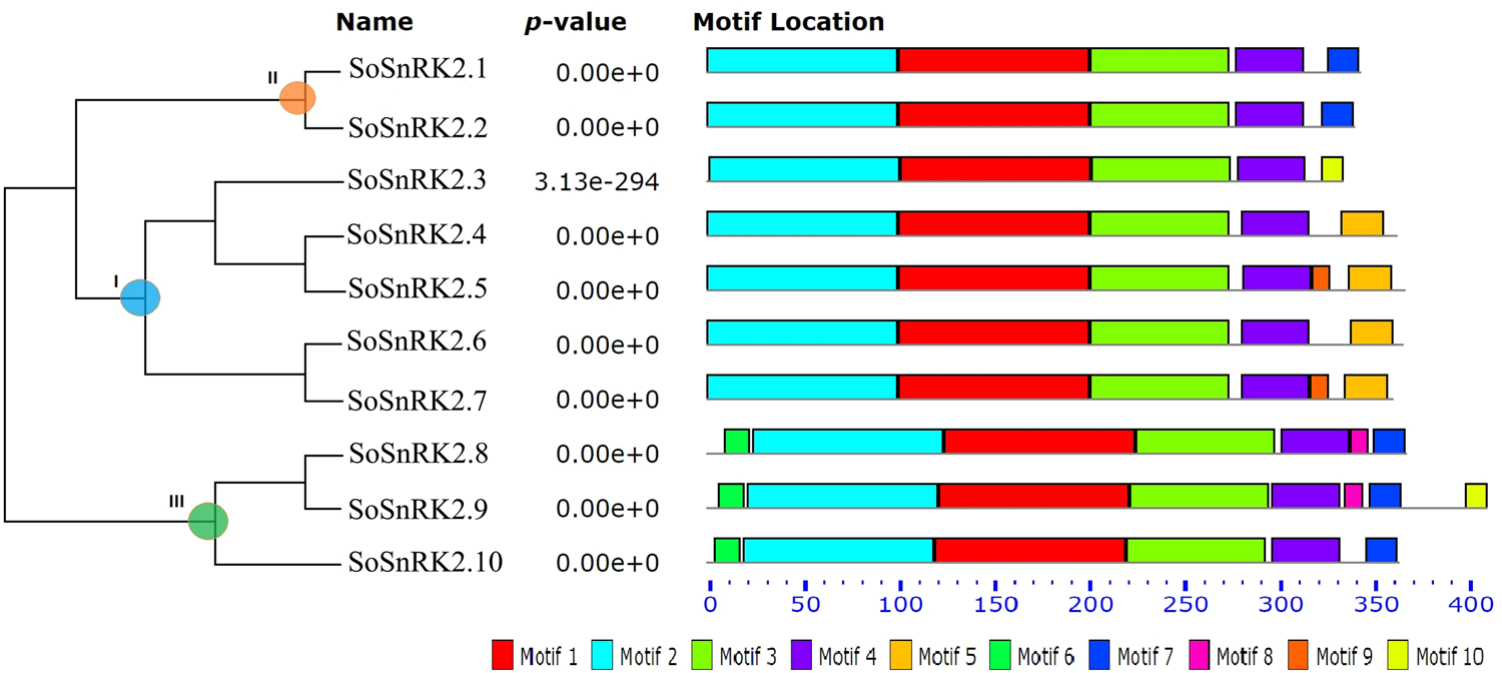
Diagrammatic structure of *SoSnRK2* genes. The unrooted phylogenetic tree was obtained via full length amino acid alignment of all SoSnRK2 proteins in sugarcane, and was constructed with the maximum likelihood (ML) method. Bootstrap values were calculated with 1000 replicates using MEGA7.0. The motifs were identified using the MEME program with an optimum motif width of 10–100 bp and a maximum motif number set at 10.

The previous analyses have showed all SnRK2s have an N-terminal conserved catalytic domain similar to SNF1/AMP kinases and a short C-terminal regulatory domain which is not highly conserved. Using the MEME motif research tool, 10 motifs were identified in SoSnRK2s (Fig. 3). Motif 1 and motif 2 were contained in the N-terminal of all genes, suggesting that they belong to the protein kinase domain. In addition, motif 3 and motif 4 could be found in all genes, and the motifs of the C-terminal were more similar within groups than between groups. Motif 8 was only present in group III, suggesting its involvement in ABA response.

**Figure 4:**
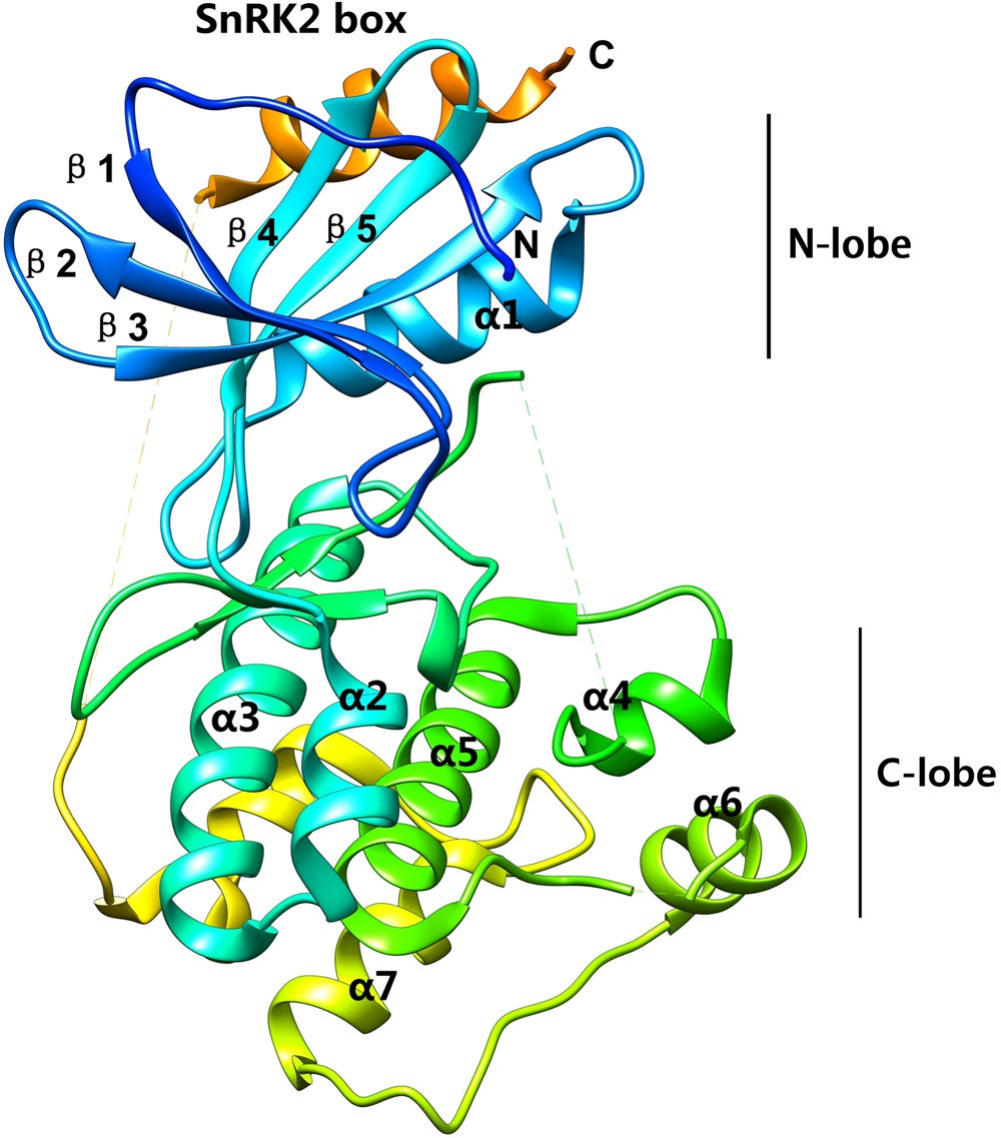
Predicted secondary structure of *SoSnRK2* genes. The SnRK2 boxes are highlighted in orange. Parts that are not resolved in the structures are the C-terminal 44 residues harboring the ABA box and the segments indicated by dotted lines.

To better understand the conserved amino acid distributions and spatial structural evolution of SoSnRK2s, we established a three delimitation structural model for SoSnRK2s using the ConSurf web server on the basis of AtSnRK2.3 (Fig. 4). In the putative structural model of the SoSnRK2s, the conserved region was located in the inner part, while most residues of SoSnRK2s were located in the cover, at the junction of the larger C-terminal lobe. The smaller N-terminal lobe resides on the catalytic cleft and contains the binding sites for substrate and ATP. A flexible hinge that allows kinase domains to adopt two alternative ensembles of conformations connects both lobes: open conformations that are indicative for inactive kinases and closed conformations that are adopted by active kinases. Overall, the protein structure and alignment results demonstrated the high evolutionary conservation of the SnRK2s.

### Organ specific expression of *SoSnRK2s*

**Figure 5:**
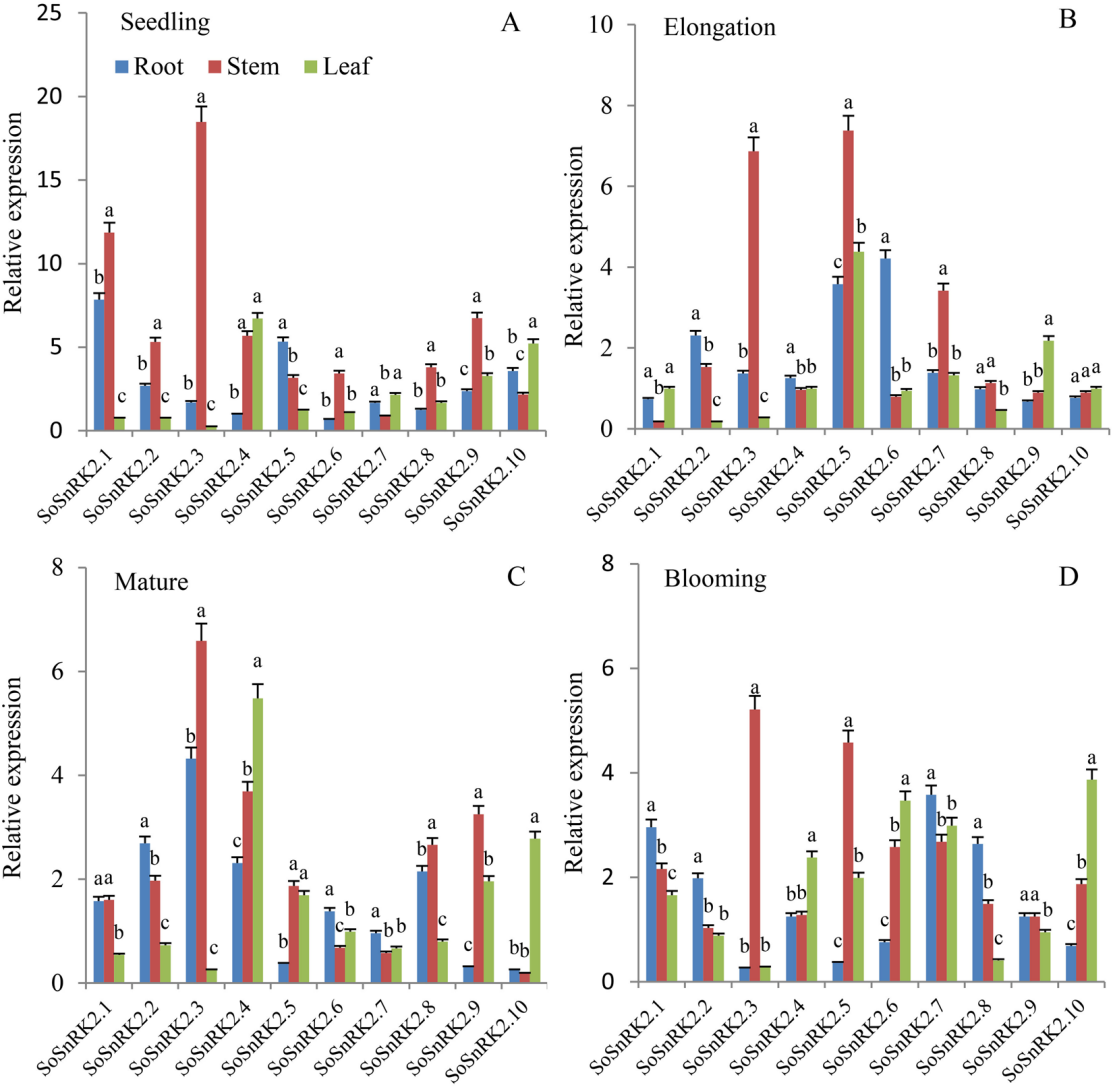
Organ specific expression patterns of *SoSnRK2* genes. The expression profiles of *SoSnRK2* genes in roots, stems, and leaves were examined under different growth stages. Relative expressions of *SoSnRK2* genes were analyzed via qRT-PCR and the relative transcript levels were quantified against *SoGAPDH* transcript levels using the 2^−ΔΔCt^ method. Data are mean ± SE from three biological replicates. Bars superscripted by a different letter indicate that gene expression significantly differed at 0.05 probability of the same gene in different organs.

**Figure 6:**
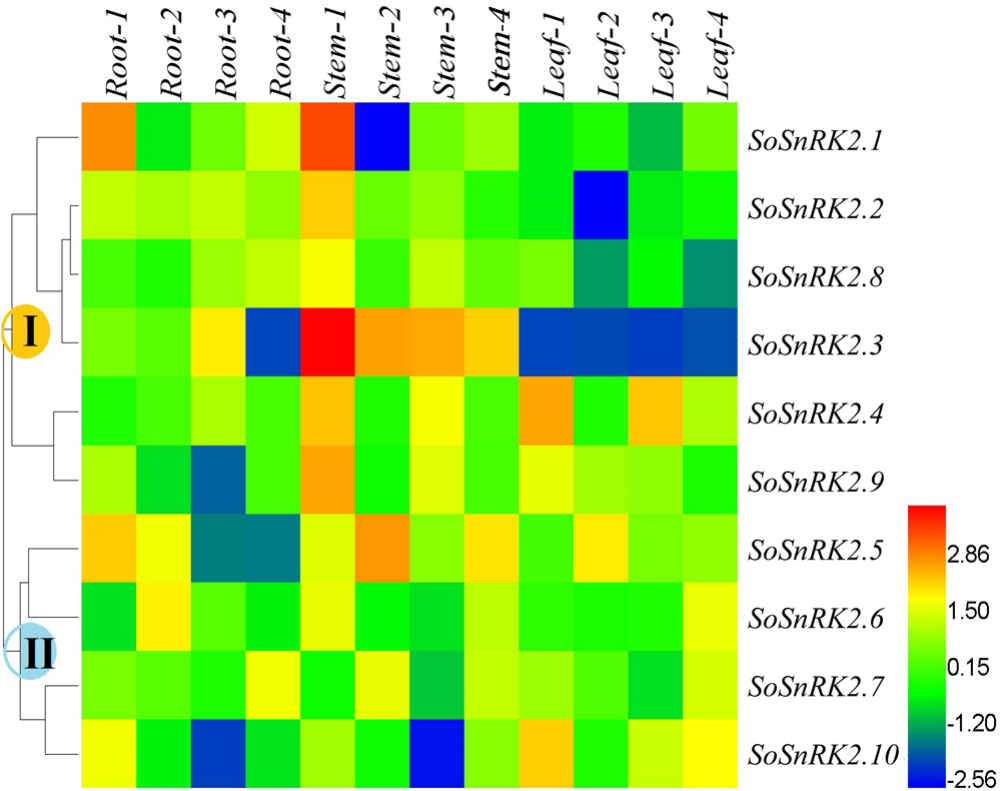
Organ expression patterns of *SoSnRK2* genes during the life cycle in sugarcane plant. The hierarchical cluster displays the expression profile for ten *SoSnRK2* genes at different growth stages. Root/Stem/Leaf-1/2/3/4 represents the gene expression in root/stem/leaf under seeding/elongation/mature/blooming stage, respectively. The color bar at the right side represents log2 expression values: Blue represents low expression, green medium expression and red high expression.

The expression profiles of *SoSnRK2s* in roots, stems and leaves were examined at different developmental stages (Fig. 5). Eight genes (except *SoSnRK2*.*7/2*.*10*) had globally high expression values in stem tissue at the seedling stage (Fig. 5A), and all genes had similar expression levels throughout all tested organs, besides *SoSnRK2*.*3*/*2*.*7/2*.*8* in leaves and *SoSnRK2*.*1* in stems at the elongation stage (Fig. 5B). At the mature stage, *SoSnRK2*.*1*/2.2/*2*.*3*/*2*.*8* had high expression values in roots and stems (Fig. 5C), and these genes were assigned to the same group. However, the expression of *SoSnRK2*.*3* in roots and leaves, *SoSnRK2*.*8* in leaves, as well as *SoSnRK2*.*5*/*2*.*6*/*2*.10 in roots was relatively lower than in other organs at the blooming stage (Fig. 5D). A hierarchical cluster was generated according to the gene expression patterns and ten *SoSnRK2s* could be distinctly classified in two groups (Fig. 6): Six genes (*SoSnRK2*.*1/2*.*2/2*.*3/2*.*4/2*.*8/2*.*9*) were assigned to Group I, of which showed low transcript accumulations in the analyzed leaf tissues. The four remaining genes were assigned to Group II, which exhibited preferential low expression signals in root or stem tissues.

### Transcript changes of *SoSnRK2s* under abiotic stresses

**Figure 7:**
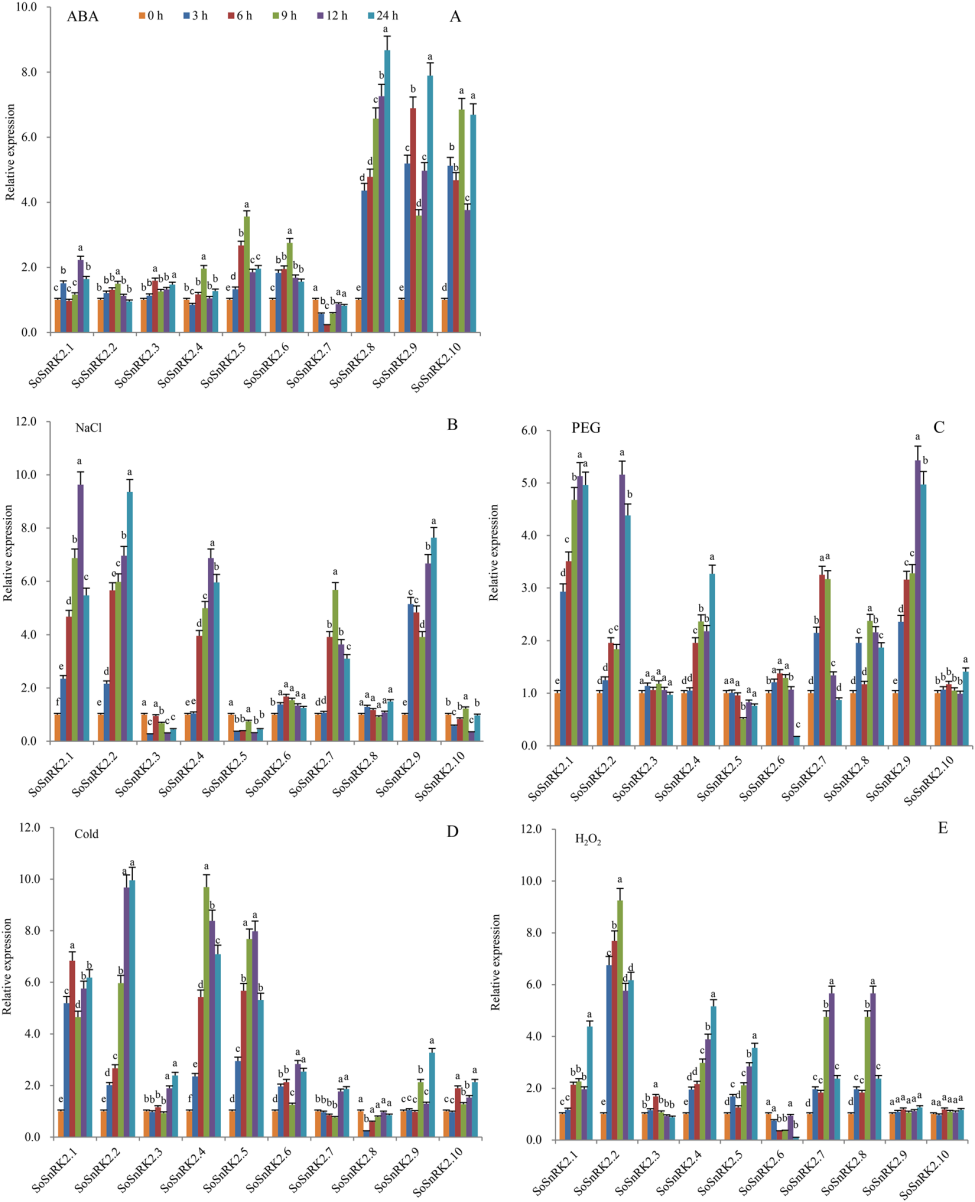
Expression levels of *SoSnRK2* genes following short term abiotic stresses of ABA, PEG, NaCl, cold, and H_2_O_2_ treatments. The ten stress-inducible *SoSnRK2* genes were differently expressed in the leaves in response to abiotic stressors and their transcript levels were quantified against *SoGAPDH* transcript levels using the 2^−ΔΔCt^ method. Gene expression at 0 h was used as control. Data are mean ± SE from three biological replicates. Bars superscripted by a different letter indicate that gene expression was significantly different at the 0.05 probability of the same gene at different sampled time.

*SnRK2s* have been identified to widely participate in abiotic stress responses. In this study, transcript changes of *SoSnRK2s* under different stress were analyzed and showed that all *SoSnRK2s* were responsive to at least two stress signals (Fig. 7). The induced intensity of all group III gene members was higher than those of the other two groups under ABA treatment (Fig. 7A). In detail, *SoSnRK2*.*8* was gradually induced throughout treatment time and taking the highest expression among all *SoSnRK2s*. Both *SoSnRK2*.*9* and *SoSnRK2*.*10* had two peak expression values at 6/24 h, and 9/24 h, respectively. The group II member *SoSnRK2*.*2* was slightly induced at 12 h and *SoSnRK2*.*1*/*2*.*2* expression remained stable during the remaining treatment time. The group I members *SoSnRK2*.*5*/*2*.*6* had relatively higher expression values, while *SoSnRK2*.*3/2*.*4* showed almost no change, but *SoSnRK2*.*7* showed significantly expression repressed by ABA.

As for salt stress, five genes (*SoSnRK2*.*1*/*2*.*2*/*2*.*4*/*2*.*7*/*2*.*9*) showed significant up-regulation, while three genes (*SoSnRK2*.*3/2*.*5/2*.*10*) showed down-regulation. The remaining two genes (*SoSnRK2*.*6/2*.*8*) had no significant changes (Fig. 7B). Under PEG6000 treatment, six genes showed increased expressions, whereas the remaining four genes (*SoSnRK2*.*3/2*.*5/2*.*6/2*.*10*) showed almost no change (Fig. 7C). At low temperature, the group I members *SoSnRK2*.*4/2*.*5* were significantly induced, while *SoSnRK2*.*3*/*2*.*7* were strongly reduced up to 9 h, then slowly rebounded from 12 to 24 h. The expression of group III members *SoSnRK2*.*9*/*2*.*10* and group II members *SoSnRK2*.*1*/*2*.*2* were both induced by cold stress (Fig. 7D).

**Figure 8:**
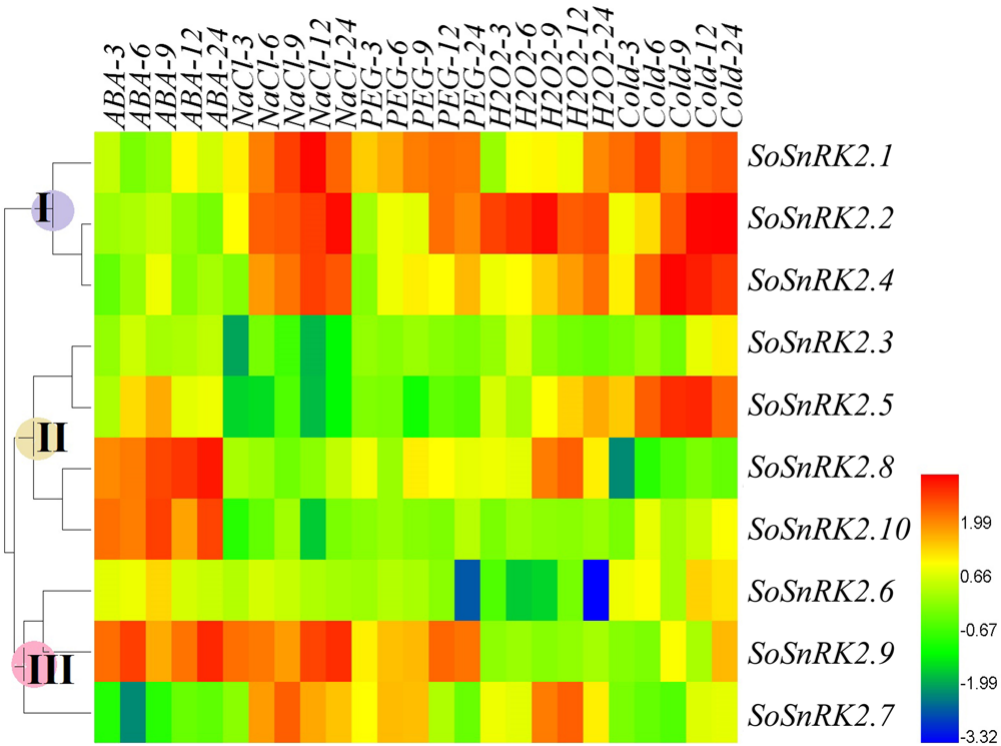
Expression patterns of *SoSnRK2* genes following short term abiotic stresses of ABA, PEG, NaCl, cold, and H_2_O_2_ treatments. The number 3/6/9/12/24 behind each treatment represents sampling time. The color bar at the right side represents log2 expression values: Blue represents low expression, green medium expression and red high expression.

H_2_O_2_ molecules play important roles in regulating plant developmental processes and signaling networks are involved in responses to a wide range of biotic and abiotic stresses. Here, the responses of *SoSnRK2s* to H_2_O_2_ stress were tested and the results indicated that six genes (*SoSnRK2*.*1/2*.*2/2*.*4/2*.*5/2*.*7/2*.*8*) that belong to different groups were induced by H_2_O_2_. *SoSnRK2*.*6* showed decreased expression, while the three remaining genes had no significant changes (Fig. 7E). A hierarchical cluster was generated according to the gene expression patterns and ten *SoSnRK2s* could be distinctly classified in three groups (Fig. 8): *SoSnRK2*.*1/2*.*2/2*.*4* belonged to Group I, which showed high transcript accumulations in all abiotic stresses except in ABA treatment. Group II consists of four genes, of which *SoSnRK2*.*8* */2*.*10* were significantly induced by ABA, while *SoSnRK2*.*5* was induced by cold stress. Group III contains three genes (*SoSnRK2*.*6/2*.*7/2*.*9*), which strongly induced by salt and drought stress.

### Protein kinase activity triggered by abiotic stresses

**Figure 9:**
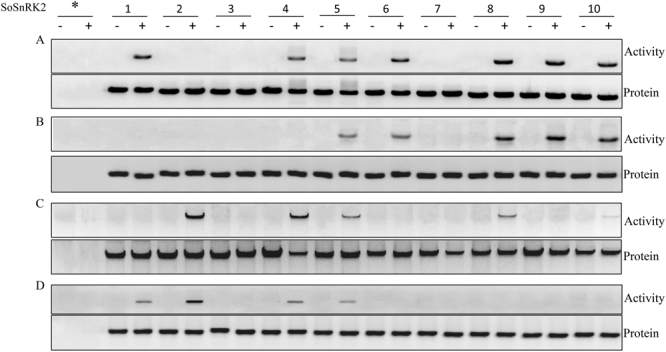
SoSnRK2 activation in response to NaCl (**A**), ABA (**B**), H_2_O_2_ (**C**) and cold (**D**) stress. *Arabidopsis* protoplasts were transiently transformed with the empty vector (*) or the expression vector for each SoSnRK2. Protoplasts were transferred to isosmotic medium [400 mOsm (−)] or NaCl salt stress [1000 mOsm (+)] for 15 min (**A**); For ABA (B) and oxidative stress (**C**), protoplasts were incubated with 100 µM ABA (+) or 10 mM H_2_O_2_ ( + ), or the same volume of ethanol solvent control (−) for 20 min (**C**), respectively. Protoplasts were incubated at room temperature (−) or at 4 °C (+) for 20 min for cold treatment (**D**). Number 1–10 on the head of the figure indicated the SoSnRK2.1-SoSnRK2.10. HA-tagged SoSnRK2 proteins were immunoprecipitated from protoplasts protein extracts with anti-HA antibody and analyzed by in-gel kinase assay using MBP as a substrate (*upper panel*). The expression of each kinase was monitored by immunoblotting the same extracts with anti-HA antibody (*lower panel*).

The protein kinase activity of SoSnRK2 activated by abiotic stresses was investigated using a transient expression assay. The transfected protoplasts were treated for different abiotic stresses. After immunoprecipitation with anti-HA antibody, SnRK2 activity was determined by in-gel kinase assay using MBP (myelin basic protein) as a substrate (Fig. 9). All SoSnRK2 except SoSnRK2.2/2.3/2.7 were activated by salt stress. Two activation levels can be distinguished: with a strong activation of SoSnRK2.1/2.6/2.8/2.9/2.10 than SoSnRK2.4/2.5 (Fig. 9A). By contrast with saline treatments, ABA only activated five SoSnRK2 (Fig. 9B), of which SoSnRK2.8/2.9/2.10 were more significantly induced by ABA than SoSnRK2.5/2.6. As for H_2_O_2_ treatment, a very strong activation of SoSnRK2.2, a strong activation of SoSnRK2.4, and a lower activation of SoSnRK2.5 and SoSnRK2.8 were observed (Fig. 9C). Four SoSnRK2 were activated by cold stress (Fig. 9D), the expression intensity of SoSnRK2.2 looked similar as compared to H_2_O_2_ treatment. The other three (SoSnRK2.1/2.4/2.5) were only very slightly activated by cold when contrary to the high activation of SoSnRK2.2.

## Discussion

To withstand unfavorable environmental conditions, plants have acquired physiological and biochemical strategies that regulate the expression of transcription factors. These factors can be involved in controlling multiple genes from various pathways or by overexpressing genes related to stress signal perception and transduction^37^. The SnRK2 contains a small plant specific protein group that can be activated by environmental stress^10–13^. The first *SnRK2* gene was identified from an ABA treated wheat embryo cDNA library^14^ and to date, most *SnRK2*s in model plant species such as *Arabidopsis*, maize, and rice have been characterized^9,10,18,25,26^. Although gene evolutionary features and conserved structures have been studied in these species, various expression patterns and functional diversities of SnRK2s deserved specific attention^38^.

Here, ten *SoSnRK2*s were identified from a sugarcane full-length cDNA library. Phylogenetic and gene structure analyses indicated similarity between SoSnRK2s to counterparts in *Arabidopsis*, *Oryza sativa*, *Brachypodium distachyon*, and *Sorghum bicolor*, implying that the SnRK2 kinase evolved prior to the divergence of dicots and monocots. Gene duplication not only provided abundant raw genetic material, but might also have produced the bulk of genetic variation that has enabled plants to adapt to natural selection pressures during evolution^39^. The data generated in this study indicated that successive rounds of gene duplication have occurred in the *SoSnRK2* family, resulting in the rapid expansion of gene members. It has been reported that *SnRK2s* could be divided into three groups of which group III being the oldest group^40^. The phylogenetic relationships of all SnRK2 members from various plants and the pairwise sequence divergence for the protein coding region of *SoSnRK2s* are supporting previous work.

The plant SnRK2 contains two typical domains: a highly conserved N-terminal protein kinase and a C-terminal variable adjusting domain. Extensive evidence indicated that the C-terminal domain plays a role in the functional diversity of SnRK2s^3,9,41^. Conserved motif analysis has showed an uneven distribution of ten motifs in SoSnRK2s sequences. Among these, motifs 1, 2, 4, and 6 can be found in all members, motif 5 and 9 were only present in subclasses I, motif 7 was specific to groups II and III, and motif 3 and 8 were unique to subclass III, suggesting that they might contribute to the functional specificity of corresponding groups. However, further studies are required on the motif-exchange experiment using protein interaction assays. SnRK2s are monomeric plant-specific Ser/Thr protein kinases with a molecular weight of approximately 40 kDa^42^. So far, the group III members *AtSnRK2*.*2*/*2*.*3/2*.*6* have been systematically studied in *Arabidopsis*, and their structural profiles have been well characterized^10,43^. Based on the amino acid sequence alignment and structural profile of AtSnRK2.3/2.6 and SoSnRK2s, some of the key segments near the N-terminal have been identified to contribute to basal activities (including ATP binding loop, ATP binding site, proton acceptor activate site, activation loop and phosphoserine site). Furthermore, the α-helix and β-bridge were highly conserved. Sequence segments of the SnRK2 box, ABA box, and functional domains (domain I and domain II) that can be found near the C-terminal, were highly diversified in each sequence, which is in accordance with previous research of the functional diversity of SnRK2s known to be closely related to their C-terminal^3,9,41^.

Plant stress tolerance is achieved by a complex signal transduction pathway by both ABA dependent and ABA independent resistance mechanisms. Previous work has demonstrated that the SnRK2s has specifically involved in respond to various types of stresses and that individual members have acquired different regulatory properties, including ABA responsiveness^22–25^. The expression profiles of *SnRK2s* have been studied in *Arabidopsis* and rice, which indicated the group III members of *SnRK2*s to be essential components of ABA signaling and highly induced by ABA treatment^3,17,38^. In the present study, a comprehensive expression analysis of *SoSnRK2s* showed that all of them significantly respond to several stresses and signal molecules. When treated with ABA however, not only *SoSnRK2*.*7*, *SnRK2*.*8*, and *SnRK2*.*9* (members of group III), but also *SoSnRK2*.*5* and *SoSnRK2*.*6* (members of group II) were induced by ABA in our study (Similar kinase activation profiles were observed when protoplasts were exposed to ABA treatment). These results differ from those previously reported for *Arabidopsis* and rice, in which only group III members (*AtSnRK2*.*2/2*.*3/2*.*6* in *Arabidopsis* and *OsSAPK8*-*10* in rice) were strongly induced by ABA^3,17,38^, and indicating the complicated ABA-dependent signal transduction pathway in sugarcane. However, further experiments using ABA deficient or insensitive mutants are needed to conclude on the ABA dependence of SoSnRK2s activation. In addition, *SoSnRK2*.*7* (a member of group II) was significantly repressed by ABA treatment, suggesting its special role in the response to ABA signaling. *SoSnRK2*.*1*, *SoSnRK2*.*2*, and *SoSnRK2*.*4* responded to all tested treatments except for ABA, but *SoSnRK2*.*1* and *SoSnRK2*.*2* showed more than 5-fold expression changes upon salt, drought and cold stress. In the transient expression assay, only SoSnRK2.4 was activated by all tested treatments except for ABA. This kind of activation difference occurred maybe due to the special signaling roles of SoSnRK2 kinases in different stress stages. SoSnRK2.5, which was the only SnRK2 to be activated by the four signals tested in the transient expression assay, and indicating the cross-talks between hyperosmotic stress at the kinase level. However, SoSnRK2.3 and SoSnRK2.7 were not activated by any of the four stresses tested, this kind of activation occurred maybe due to involvement of these two genes in another signal transduction pathway.

It has been reported that *SnRK2s* are involved in the regulation of plant growth and development. For example, *AtSnRK2*.*4* and *AtSnRK2*.*10* (members of group I) in *Arabidopsis*, were involved in root growth and architecture under saline conditions^44^. In addition, three members of group III (*AtSnRK2*.*2*, *AtSnRK2*.*3*, and *AtSnRK2*.*6*) were shown to play roles in seed development, germination, dormancy, seedling growth, and flowering time^10–13^. Here, the expression patterns of *SoSnRK2* genes were examined in various tissues and diversification was found, indicating functional diversity of *SoSnRK2s* in sugarcane growth and development. In summary, the organ expression patterns and the expression profiles of *SoSnRK2s* in response to abiotic stresses and to signal molecules indicated both conserved as well as diverse biological functions of *SnRK2* genes within the plant kingdom.

In conclusion, we isolated and characterized ten *SoSnRK2* genes from sugarcane and elucidated their structure, conserved motifs and their potential involvement in terms of gene expression in response to abiotic stresses and stress signals, as well as to developmental signals. An assessment of expression and co-expression patterns revealed conservation and diversification between *Arabidopsis* and the sugarcane *SnRK2* gene family, both in terms of sequences and functions. This detailed baseline information will enable further understanding of the function and molecular mechanisms of *SoSnRK2s* during plant stress response and can support the breeding of sugarcane in future.

## Materials and Methods

### Plant materials and treatments

A single bud of the sugarcane variety GT21 was initially raised in sand culture. For abiotic stress treatments, 21-day-old seedlings were transplanted into plastic pots, containing 6 L modified Hoagland solution^45^. The solution was aerated by air pump and changed every 4 days. After 3 weeks, plants were transferred for multiple treatments. For ABA, salt stress, osmotic stress, and oxidative stress, seedlings were subjected to a Hoagland solution containing 100 µM ABA, 200 mM NaCl, 25% (*w/v*) PEG-6000 (Polyethylene glycol), and 10 mM H_2_O_2_, respectively. For cold stress, seedlings were exposed to 4 °C conditions. Leaf samples were collected at 0, 3, 6, 9, 12, and 24 h after treatment. For organ-specific expression analysis, plant growth conditions were identical to those previously described^46^. Organs including the root, stem, leaf, or spikelet were collected at seedling, elongation, mature, and blooming growth stage (Supplementary Figure S1). All samples were obtained in three biological replicates, frozen in liquid nitrogen and stored at −80 °C until use.

### Cloning of *SoSnRK2* coding sequences

Total RNA was isolated from the entire above samples, separately. Equal amount of total RNA from each sample were mixed and using for mRNA purification by oligo(dT)-cellulose (Qiagen). A full-length cDNA library was constructed with an optimized cap-trapper method^47^ and the cDNA database was generated with the 3′- and 5′-end sequencing data of the library. To obtain the *SoSnRK2* gene family coding sequences, the amino acid sequences of *SnRK2* gene family members of *Arabidopsis thaliana*, *Oryza sativa*, *Zea mays*, *Brachypodium distachyon* and *Sorghum bicolor* (Supplementary Table S2) were downloaded from Phytozome database^48^, and used as query probes to separately screen the cDNA database with tBLASTn. Sequences with maximum similarity (>99%) to query probes from the database were considered as candidate clones, and the clones corresponding to the same family member were assembly by Contig Express. Amplified primers (Supplementary Table S1) were designed according to clone sequences for further identification.

### Sequence analysis

The molecular mass and isoelectric points were predicted by ExPASy (http://expasy.org/). The motifs were identified by MEME (http://meme.sdsc.edu/meme/intro.html)^49^, with an optimum motif width of 10–100 bp and a motif maximum number set at 10. Protein subcellular localization was predicted using WoLF PSORT (http://wolfpsort.org/)^50^. Gene structure was identified via the Gene Structure Display Server (http://gsds.cbi.pku.edu.cn/) and the feathers of genes, using the EBI-Tools (http://www.ebi.ac.uk/Tools/emboss/). The phylogenetic tree was constructed by MEGA 7.0 (http://www.megasoftware.net/)^51^. Multiple sequence alignment was performed using MUSCLE (http://www.ebi.ac.uk/Tools/msa/muscle/) and colored with ESPript (http://espript.ibcp.fr/ESPript/ESPript/). The protein module was presumed by SWISS-MODEL (http://swissmodel.expasy.org/) and edited via Swiss-PdbViewer (http://spdbv.vital-it.ch/TheMolecularLevel/SPVTut/) according to AtSnRK2.3 (PDB ID: 3UJG).

### qRT-PCR analysis

The cDNA was synthesized from the total RNA using the Prime Script RT reagent Kit (TaKaRa). PCR was run using the primers presented in Supplementary Table S3 and performed in an iQ5 Real Time PCR Detection System (Bio-Rad) with a SYBR Green PCR Master Mix (Applied Biosystems). PCR conditions were 95 °C for 10 min, followed by 40 cycles at 95 °C for 10 s, 60 °C for 20 s, and 72 °C for 20 s. Melting curve analysis was conducted to confirm the specificity of the amplification. For each reaction, three technical and three biological replicates were carried out. The relative quantification of *SoSnRK2* genes to *GAPDH* was calculated by the 2^−ΔΔCt^ method^52^. The log2 values of gene expression fold changes compared to the corresponding control in each experiment were used to obtain heatmap that were generated by HemI^53^.

### Gateway cloning of *SoSnRK2*

The ORF of *SoSnRK2* genes were amplified by PCR using primers (Supplementary Table S4) containing *attB1*and *attB2* sequences that supplied by Invitrogen. The stop codon of reverse primers was removed to allow C-terminal fusions. The BP reactions LR reactions were carried out according to the manufacturer’s instructions (Invitrogen) using pDONR207 and pGreen-HiA-GW as entry and destination vector, separately.

For the construction of destination vector *pGreen-HiA-GW*, the Gateway conversion cassette C was inserted in the BamHI site of the intron-tagged HA-epitope cassette of pPILY vector after filling in the site with Klenow polymerase I. The resulting cassette was inserted in the KpnI site of pGreen0129 (www. pGreen.ac.uk), this construction allows the expression of HA-tagged SoSnRK2s (pGreen-HiA-SnRK2) under the control of a 35 S promoter.

### Protoplasts preparation and transient expression assays

*Arabidopsis* cell cultures were used 3 days after subculturing at 33% (v/v), protoplasts preparation and transient expression assays were essentially performed as previously described^54^. Typically, 1.5 × 10^6^ protoplasts were mixed with 25 µg of plasmid DNA (pGreen-HiA-SoSnRK2) and three volumes of PEG solution [25% (w/v) PEG-6000, 450 mM mannitol, 100 mM Ca(NO_3_)_2_]. After a 15 min incubation in the dark, the transfection mixture was washed with 275 mM Ca(NO_3_)_2_, then 250 × g centrifugation for 5 min. Protoplasts were resuspended in 1 ml of JPL-A and incubated in the dark for 15 h before use^54^.

### Protoplast treatments

For osmotic stresses, protoplasts were centrifuged (250 × g for 5 min) and resuspended in the same volume of isoosmotic medium (JPL-A, 400 mOsm) or hyperosmotic medium containing NaCl (JPL-A supplemented with 350 mM NaCl, 1000 mOsm) for 15 min. For cold treatment, protoplasts were incubated for 20 min at 4 °C or 25 °C for the control. For ABA and oxidative stress, protoplasts were incubated for 20 min with 100 µM ABA or 10 mM H_2_O_2_, respectively. All treatments were executed in three replicates. Protoplasts were centrifuged at 250 × g for 5 min to stop treatments, and then the pellet was frozen in liquid nitrogen and stored at −80 °C.

### Protein extraction, immunoblotting, immunoprecipitation and in-gel kinase assay

Protein extraction from protoplasts and immunoblotting were essentially performed as previously described^24^. The immunopurification was performed as described by Boudsocq *et al*.^54^ with some modification. 150 µg proteins was incubated with either 2.0 µg polyclonal anti-HA antibody (Sigma) or 20 µl of 50% monoclonal anti-HA-agarose antibody (Sigma) in immunoprecipitation buffer. Incubation was performed for 4 h, and 30 µl of 50% protein A-Sepharose CL-4B (Sigma) were added during the last hour when polyclonal antibodies were used. The immunoprecipitate was washed four and two times in immunoprecipitation and kinase buffer (20 mM Tris-HCl pH 7.5, 12 mM MgCl_2_, 2 mM EGTA, 2 mM DTT, 0.1 mM orthovanadate), respectively. The immunoprecipitation proteins were separated on 12% SDS-PAGE gels embedded with 0.2 mg/ml MBP. The gels were treated for protein renaturation as previously described^55^. For the activity, the gels were preincubated for 30 min at room temperature in kinase activity buffer (40 mM HEPES, pH 7.5, 2 mM DTT, 20 mM MgCl_2,_ 1 mM EGTA, 0.1 mM orthovanadate). Phosphorylation was performed for 1 h in 8 ml of the same buffer supplemented with 25 μM cold ATP and 2.9 MBq of [γ-^33^P] ATP per gel. Gels were washed five times over a period of 5 h in stop buffer [1% (w/v) Na_2_H_2_P_2_O_7_, 5% (v/v) trichloric acid]. The protein kinase activity was detected on the dried gels by the Storm imaging system (Amersham Biosciences, Uppsala, Sweden).

## Electronic supplementary material

Supplementary information
