# Supplementary material for: Molecular Characterization and Co-expression Analysis of the SnRK2 Gene Family in Sugarcane (Saccharum officinarum L.)
Source: Sci Rep. 2017 Dec 15;7:17659. doi: 10.1038/s41598-017-16152-4 (PMC5732291; doi:10.1038/s41598-017-16152-4)
Supplement: Supplementary file 1 — Supplementary information [file 41598_2017_16152_MOESM1_ESM.pdf]

Molecular Characterization and Co-expression Analysis of the *SnRK2* Gene Family in Sugarcane (*Saccharum officinarum* L.)

Changning Li[1, 2], Qian Nong[2], Jinlan Xie[2], Zeping Wang[1, 2], Qiang Liang[2], Manoj Kumar Solanki[2], Mukesh Kumar Malviya[2], Xiaoyan Liu[2], Yijie Li[2], Reemon Htun[2], Jiguang Wei[1] & Yangrui Li[1, 2]

[1]College of Agriculture, State Key Laboratory of Conservation and Utilization of Subtropical Agro-bioresources, Guangxi University, Nanning, Guangxi 530004, China.

[2]Key Laboratory of Sugarcane Biotechnology and Genetic Improvement (Guangxi), Ministry of Agriculture, Guangxi Key Laboratory of Sugarcane Genetic Improvement, Sugarcane Research Center of Chinese Academy of Agricultural Sciences, Nanning, Guangxi 530007, China.

Correspondence and requests for materials should be addressed to J. W. (email: jiguangwei@gxu.edu.cn) or Y. L. (email: liyr@gxaas.net)

Supplementary information

Table S1 The primer sequences used for cDNA cloning of *SoSnRK2s*

Table S2 The information of *SnRKs* in queries

Table S3 Gene-specific primer sequences for qRT-PCR

Table S4. Table S4. Oligonucleotides used for *SoSnRK2* cloning into pDONR207

Figure S1 Different development stages of sugarcane

Figure S2 Protein sequence alignment of SoSnRK2s and SnRK2s from other plant species

Table S1 The primer sequences used for cDNA cloning of *SoSnRK2s*.

Gene	Forward primers(5'-3')	Reverse primers(5'-3')	Tm (℃)	Production length (bp)	Accession number	Amino acids	MW(kDa)	pI
SoSnRK2.1	ATGGAGCGGTACGAGGTGATCAGGG	TCACACTGCACAAACGAAGTCGCCG	58	1029	KT961033	342	38.8	5.20
SoSnRK2.2	ATGGAGAGGTACGAGGTGATCAAGG	TCACAAGGCACATACAAAGTCGCCG	58	1020	KT961034	339	38.5	5.74
SoSnRK2.3	ATGGAGGAGAGGTACGAGGCGCT	TCAGTAGGTGTCATCAGCGTCTG	59	1002	JQ292841	333	37.8	5.54
SoSnRK2.4	ATGGATAAGTACGAGGCGGTGCG	TCATATGTGAAGAGCGTCCAAACGG	58	1086	KX867812	361	42.3	5.96
SoSnRK2.5	ATGGACAAGTACGAGCCCGTTCGT	TCAGATTTGGAGCCGGCTCATGTCG	60	1098	KX867813	365	41.9	6.51
SoSnRK2.6	ATGGAGAAGTACGAGCTGCTCAAG	TCACTTTATCAGGTGTTGAAAATCA	57	1095	KX867814	364	41.9	5.65
SoSnRK2.7	ATGGAGAAGTATGAGCTGCTCAAG	TCAGCTGATATGAAACTCCCCGCTG	60	1080	KX867815	359	41.1	5.89
SoSnRK2.8	ATGGCAGCGCCGGCGCCGGATCGGG	TCACATTGCGTACACAATCTCACCG	55	1101	KT961035	366	41.4	4.81
SoSnRK2.9	ATGGCGAGGACGCCGGCAGCGG	TCACCATGCCACAGCTTGAACAT	56	1227	KX867816	408	45.4	4.80
SoSnRK2.10	ATGGACCGGGCGGCGCTCACCGTGG	TCAAATAGCAAACCGATTCTCCCCA	56	1089	KX867817	362	40.8	4.90

Table S2 The information of *SnRKs* in queries

Subfamily	*Arabidopsis thaliana*		*Oryza sativa*		*Brachypodium distachyon*		*Sorghum bicolor*	
	**Gene name**	**Gene ID**	**Gene name**	**Gene ID**	**Gene name**	**Gene ID**	**Gene name**	**Gene ID**
SnRK2	*AtSRK2.1*	At5g08590	*OsSAPK1*	Os03g0390200	*BdSnRK2.1*	KJ850308	*SbSnRK2.1*	Sb001G350700
	AtSRK2.2	At3g50500	*OsSAPK2*	Os07g0622000	*BdSnRK2.2*	KJ850309	*SbSnRK2.2*	Sb002G379400
	AtSRK2.3	At5g66880	*OsSAPK3*	Os10g0564500	*BdSnRK2.3*	KJ850310	*SbSnRK2.3*	Sb001G294400
	AtSRK2.4	At1g10940	*OsSAPK4*	Os01g0869900	*BdSnRK2.4*	KJ850311	*SbSnRK2.4*	Sb003G370100
	AtSRK2.5	At5g63650	*OsSAPK5*	Os04g0691100	*BdSnRK2.5*	KJ850312	*SbSnRK2.5*	Sb006G279100
	AtSRK2.6	At4g33950	*OsSAPK6*	Os02g0551100	*BdSnRK2.6*	KJ850313	*SbSnRK2.6*	Sb004G173500
	AtSRK2.7	At4g40010	*OsSAPK7*	Os04g0432000	*BdSnRK2.7*	KJ850314	*SbSnRK2.7*	Sb006G083000
	AtSRK2.8	At1g78290	*OsSAPK8*	Os03g0764800	*BdSnRK2.8*	KJ850315	*SbSnRK2.8*	Sb001G078800
	AtSRK2.9	At2g23030	*OsSAPK9*	Os12g0586100	*BdSnRK2.9*	KJ850316	*SbSnRK2.9*	Sb008G147000
	AtSRK2.10	At1g60940	*OsSAPK10*	Os03g0610900	*BdSnRK2.10*	KJ850317	*SbSnRK2.10*	Sb001G168400

Table S3 Gene-specific primer sequences for qRT-PCR

Gene	Forward primers(5'-3')	Reverse primers(5'-3')	Tm (℃)	Production length (bp)
SoSnRK2.1	CTTCCGCAAGACGATCACTAGAATACT	CCAACGAATATCCGAGACAGCAGAT	60	105
SoSnRK2.2	ATACATTGCTCCAGAGGTCCTTGCTA	CGCTGAGAATCCGAGTAAGTGTCTT	60	164
SoSnRK2.3	GAGATCATGCGGATCGTTCAGGAG	GTGTCATCAGCGTCTGCCTCTTC	60	125
SoSnRK2.4	GAAGAACCTACCACGGGAACTCA	TTCATCATCCGAATACTCACTGCT	60	184
SoSnRK2.5	TGCAGCACCGAGGACTTGGAGTAG	TGCAGCACCGAGGACTTGGAGTAG	60	97
SoSnRK2.6	CAAGAAGGACAACAGTGCCCACA	AAAGCCAGCCACTTGAGTAGATGA	60	112
SoSnRK2.7	TATGTGATGCTGGTCGGTGGGTA	AGTTCCTTGCAGTCTTGGGATACG	60	137
SoSnRK2.8	CCACCATACCACCTGCCTGTTCT	ACAATCTCACCGCTGCTGTCAAC	60	124
SoSnRK2.9	TGCTGGTTGGTGGGTATCCTTTC	TGGCTCTTTATCTCAGGCATCGT	60	193
SoSnRK2.10	TCTCAGCCGAAATCTACAGTGGG	TCTGGGTCCTCAAATGGATACGC	60	152
GAPDH	GGTGAGGCTGGTGCTGACTATG	GGCAGAGATAACAACCTTCTTGGC	60	108

Table S4. Oligonucleotides used for *SoSnRK2* cloning into pDONR207

**Gene**		**Sequence of oligonucleotides forward (F) and reverse (R)**
SoSnRK2.1	F	5'-*GGGGACAAGTTTGTACAAAAAAGCAGGCT*TCACCATGGAGCGGTACGAGGTG-3'
	R	5'-*GGGGACCACTTTGTACAAGAAAGCTGGGT*CCCACTGCACAAACGAAGTCGCC-3'
SoSnRK2.2	F	5'-*GGGGACAAGTTTGTACAAAAAAGCAGGCT*TCACCATGGAGAGGTACGAGGT-3'
	R	5'-*GGGGACCACTTTGTACAAGAAAGCTGGGT*CCCAAGGCACATACAAAGTCGCCG-3'
SoSnRK2.3	F	5'-*GGGGACAAGTTTGTACAAAAAAGCAGGCT*TCACCATGGAGGAGAGGTACGA-3'
	R	5'-*GGGGACCACTTTGTACAAGAAAGCTGGGT*CGTAGGTGTCATCAGCGTCTGCCTC-3'
SoSnRK2.4	F	5'-*GGGGACAAGTTTGTACAAAAAAGCAGGCT*TCACCATGGATAAGTACGAGGCG-3'
	R	5'-*GGGGACCACTTTGTACAAGAAAGCTGGGT*CTATGTGAAGAGCGTCCAAACGGA-3'
SoSnRK2.5	F	5'-*GGGGACAAGTTTGTACAAAAAAGCAGGCT*TCACCATGGACAAGTACGAGCCC-3'
	R	5'-*GGGGACCACTTTGTACAAGAAAGCTGGGT*CGATTTGGAGCCGGCTCATGTCGA-3'
SoSnRK2.6	F	5'-*GGGGACAAGTTTGTACAAAAAAGCAGGCT*TCACCATGGAGAAGTACGAGCT-3'
	R	5'-*GGGGACCACTTTGTACAAGAAAGCTGGGT*CCTTTATCAGGTGTTGAAAATC-3'
SoSnRK2.7	F	5'-*GGGGACAAGTTTGTACAAAAAAGCAGGCT*TCACCATGGAGAAGTATGAGCT-3'
	R	5'-*GGGGACCACTTTGTACAAGAAAGCTGGGT*CGCTGATATGAAACTCCCCGCTGG-3'
SoSnRK2.8	F	5'-*GGGGACAAGTTTGTACAAAAAAGCAGGCT*TCACCATGGCAGCGCCGGCGCCG-3'
	R	5'-*GGGGACCACTTTGTACAAGAAAGCTGGGT*CCATTGCGTACACAATCTCACCGCT-3'
SoSnRK2.9	F	5'-*GGGGACAAGTTTGTACAAAAAAGCAGGCT*TCACCATGGCGAGGACGCCGGC-3'
	R	5'-*GGGGACCACTTTGTACAAGAAAGCTGGGT*CCCATGCCACAGCTTGAACATTGGA-3'
SoSnRK2.10	F	5'-*GGGGACAAGTTTGTACAAAAAAGCAGGCT*TCACCATGGACCGGGCGGCGCT-3'
	R	5'-*GGGGACCACTTTGTACAAGAAAGCTGGGT*CAATAGCAAACCGATTCTCCCCACT-3'

Note: *attB1* and *attB2* sequences are in italic

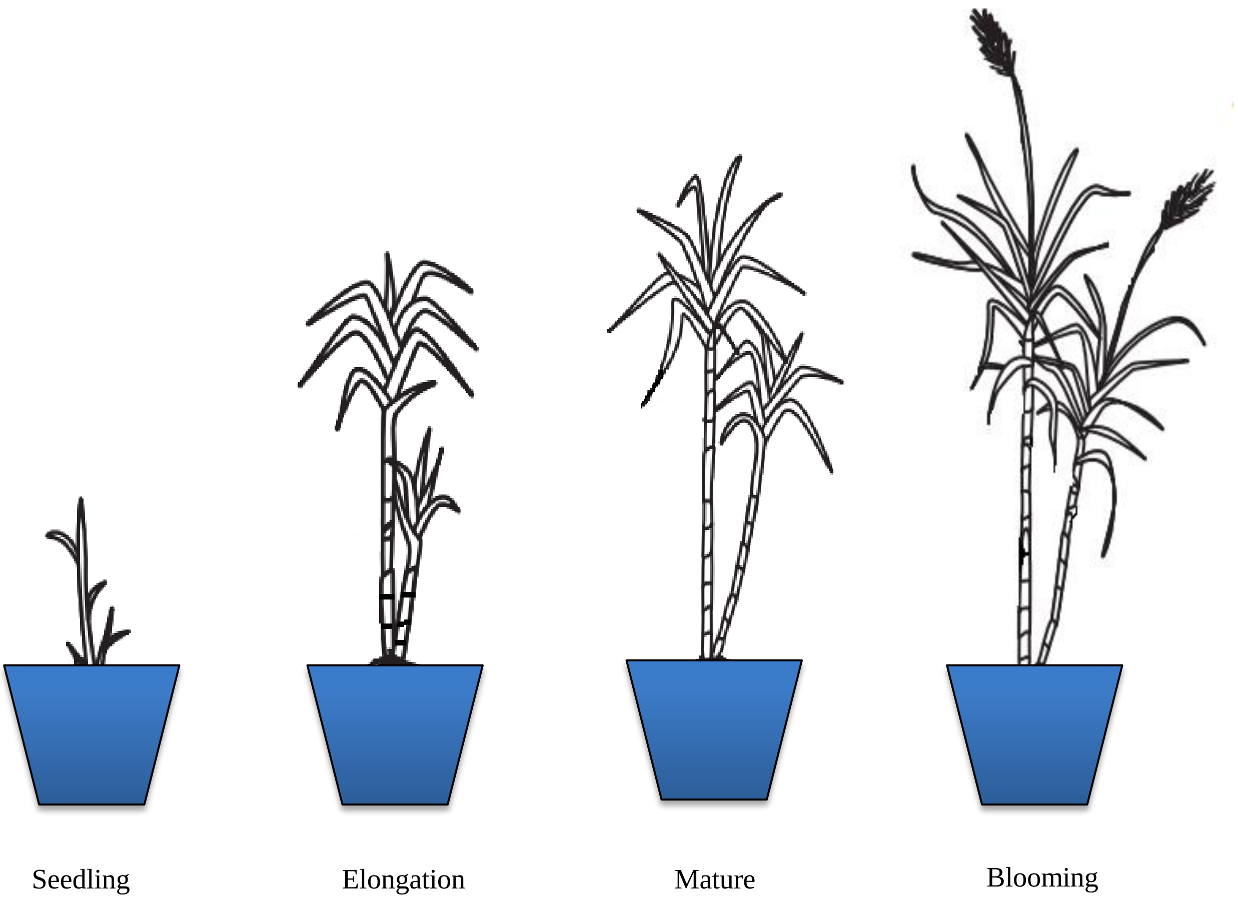

Figure S1 Different development stages of sugarcane

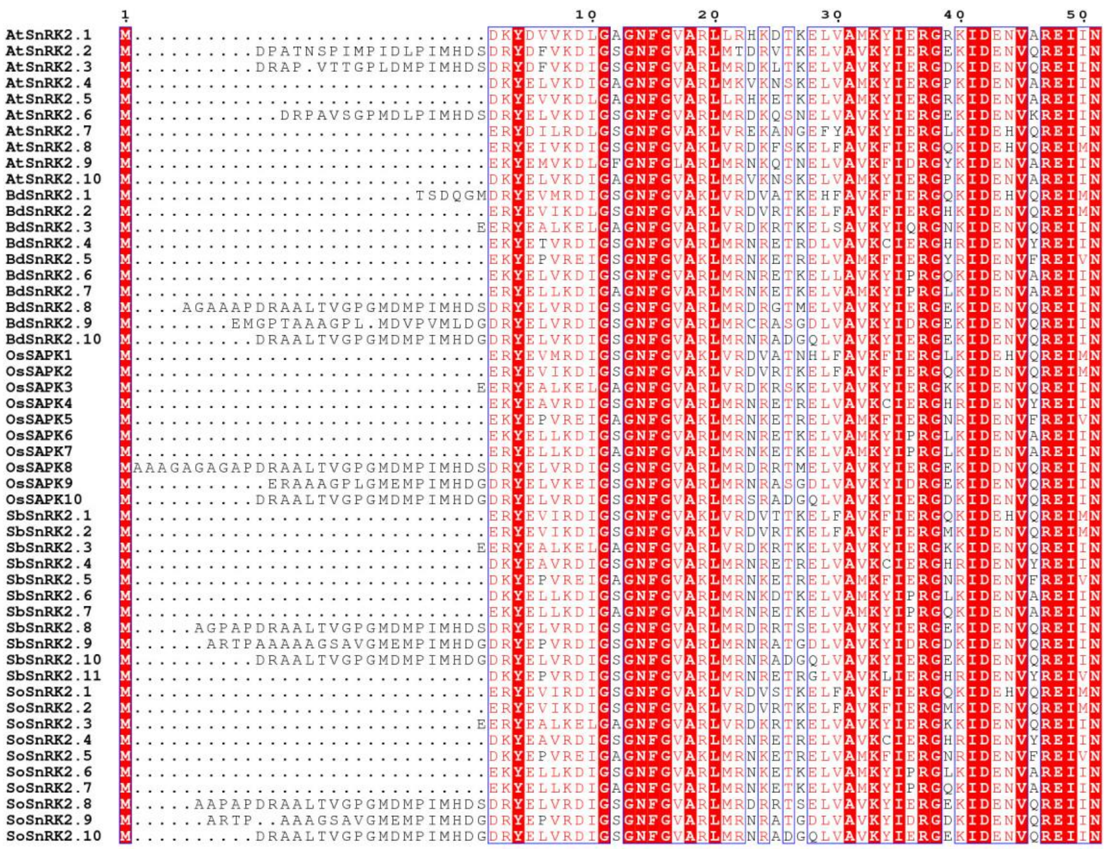

1 10 20 30 40 50
AtSnRK2.1
AtSnRK2.2
AtSnRK2.3
AtSnRK2.4
AtSnRK2.5
AtSnRK2.6
AtSnRK2.7
AtSnRK2.8
AtSnRK2.9
AtSnRK2.10
BdSnRK2.1
BdSnRK2.2
BdSnRK2.3
BdSnRK2.4
BdSnRK2.5
BdSnRK2.6
BdSnRK2.7
BdSnRK2.8
BdSnRK2.9
BdSnRK2.10
OsSAPK1
OsSAPK2
OsSAPK3
OsSAPK4
OsSAPK5
OsSAPK6
OsSAPK7
OsSAPK8
OsSAPK9
OsSAPK10
SbSnRK2.1
SbSnRK2.2
SbSnRK2.3
SbSnRK2.4
SbSnRK2.5
SbSnRK2.6
SbSnRK2.7
SbSnRK2.8
SbSnRK2.9
SbSnRK2.10
SbSnRK2.11
SoSnRK2.1
SoSnRK2.2
SoSnRK2.3
SoSnRK2.4
SoSnRK2.5
SoSnRK2.6
SoSnRK2.7
SoSnRK2.8
SoSnRK2.9
SoSnRK2.10

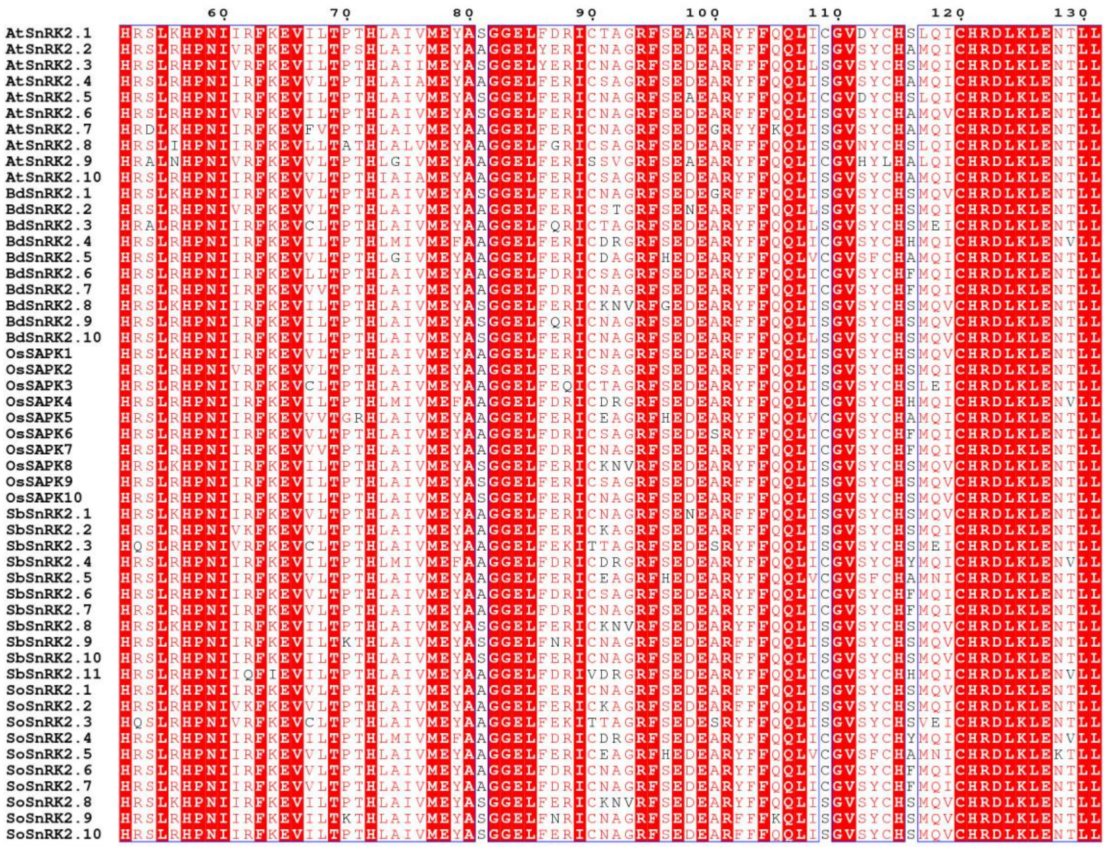

60 70 80 90 100 110 120 130
AtSnRK2.1
AtSnRK2.2
AtSnRK2.3
AtSnRK2.4
AtSnRK2.5
AtSnRK2.6
AtSnRK2.7
AtSnRK2.8
AtSnRK2.9
AtSnRK2.10
BdSnRK2.1
BdSnRK2.2
BdSnRK2.3
BdSnRK2.4
BdSnRK2.5
BdSnRK2.6
BdSnRK2.7
BdSnRK2.8
BdSnRK2.9
BdSnRK2.10
OsSAPK1
OsSAPK2
OsSAPK3
OsSAPK4
OsSAPK5
OsSAPK6
OsSAPK7
OsSAPK8
OsSAPK9
OsSAPK10
SbSnRK2.1
SbSnRK2.2
SbSnRK2.3
SbSnRK2.4
SbSnRK2.5
SbSnRK2.6
SbSnRK2.7
SbSnRK2.8
SbSnRK2.9
SbSnRK2.10
SbSnRK2.11
SoSnRK2.1
SoSnRK2.2
SoSnRK2.3
SoSnRK2.4
SoSnRK2.5
SoSnRK2.6
SoSnRK2.7
SoSnRK2.8
SoSnRK2.9
SoSnRK2.10

```
             140       150       160       170       180       190       200
AtSnRK2.1   DGSPAPLLKICDFGYS.......KSSILHSRPKSTVGTPAYIAPEVLSRREYDGKHADVWSCGVTLYVMLVGAYPFEDPN
AtSnRK2.2   DGSPAPRLKICDFGYSKVLFISLKSSVLHSQPKSTVGTPAYIAPEVLLRQEYDGKLADVWSCGVTLYVMLVGAYPFEDPQ
AtSnRK2.3   DGSPAPRLKICDFGYS.......KSSVLHSQPKSTVGTPAYIAPEVLLRQEYDGKIADVWSCGVTLYVMLVGAYPFEDPE
AtSnRK2.4   DGSPAPRLKICDFGYS.......KSSLLHSRPKSTVGTPAYIAPEVLSRREYDGKMADVWSCGVTLYVMLVGAYPFEDQE
AtSnRK2.5   DGSPAPLLKICDFGYS.......KSSILHSRPKSTVGTPAYIAPEVLSRREYDGKHADVWSCGVTLYVMLVGGYPFEDPD
AtSnRK2.6   DGSPAPRLKICDFGYS.......KSSVLHSQPKSTVGTPAYIAPEVLLKKEYDGKVADVWSCGVTLYVMLVGAYPFEDPE
AtSnRK2.7   DGSPSSHLKICDFGYS.......KSSVLHSQPKSTVGTPAYVAPEVLSRKEYNGKIADVWSCGVTLYVMLVGAYPFEDPE
AtSnRK2.8   DGSEAPRVKICDFGYS.......KSGVLHSQPKTTVGTPAYIAPEVLSTKEYDGKIADVWSCGVTLYVMLVGAYPFEDPS
AtSnRK2.9   DGSPAPRLKICDFGYS.......KSSVLHSNPKSTVGTPAYIAPEVFCRSEYDGKSVDVWSCGVALYVMLVGAYPFEDPK
AtSnRK2.10  DGSPAPRLKICDFGYS.......KSSLLHSRPKSTVGTPAYIAPEVLSRGEYDGKMADVWSCGVTLYVMLVGAYPFEDQE
BdSnRK2.1   DGSVAPRLKICDFGYS.......KSSVLHSQPKSTVGTPAYIAPEVLSRREYDGKVADVWSCGVTLYVMLVGAYPFEDPD
BdSnRK2.2   DGSEAPRLKICDFGYS.......KSSVLHSQPKSTVGTPAYIAPEVLSRREYDGKVADVWSCGVTLYVMLVGAYPFEDPV
BdSnRK2.3   DGSPTPRVKICDFGYS.......KSALLHSKPKSTVGTPAYIAPEVLSRKEYDGKVADVWSCGVTLYVMLIGSYPFEDPE
BdSnRK2.4   DGSAAPRLKICDFGYS.......KSSVLHSRPKSAVGTPAYIAPEVLSRREYDGKLADVWSCGVTLYVMLVGAYPFEDQD
BdSnRK2.5   DGSPAPRLKICDFGYS.......KSSVLHSRPKSTVGTPAYIAPEVLSRREYDGKQADVWSCGVTLYVMLVGGYPFEDSK
BdSnRK2.6   DGNPAPRLKICDFGYS.......KSSLLHSKPKSTVGTPAYIAPEVLSRREYDGKMADVWSCGVTLYVMLVGGYPFEDPD
BdSnRK2.7   DGSPAPRLKICDFGYS.......KSSILHSKPKSTVGTPAYIAPEVLSRREYDGKTADVWSCGVTLYVMLVGGYPFEDPD
BdSnRK2.8   DGSPAPRLKICDFGYS.......KSSVLHSQPKSTVGTPAYIAPEVLLKKEYDGKIADVWSCGVTLYVMVVGAYPFEDPE
BdSnRK2.9   DGSNAPRLKICDFGYS.......KSSVLHSQPKSTVGTPAYIAPEVLLKKEYDGKIADVWSCGVTLYVMLVGAYPFEDPE
BdSnRK2.10  DGSTAPRLKICDFGYS.......KSSVLHSQPKSTVGTPAYIAPEVLLKKEYDGKIADVWSCGVTLYVMLVGAYPFEDPE
OsSAPK1     DGSVTPRLKICDFGYS.......KSSVLHSQPKSTVGTPAYIAPEVLSRKEYDGKVADVWSCGVTLYVMLVGAYPFEDPD
OsSAPK2     DGSIAPRLKICDFGYS.......KSSLLHSQPKSTVGTPAYIAPEVLARKEYDGKVADVWSCGVTLYVMLVGAYPFEDPD
OsSAPK3     DGSPTPRVKICDFGYS.......KSALLHSKPKSTVGTPAYIAPEVLSRKEYDGKVADVWSCGVTLYVMLVGSYPFEDPG
OsSAPK4     DGSPAPRLKICDFGYS.......KSSVLHSRPKSAVGTPAYIAPEVLSRREYDGKLADVWSCGVTLYVMLVGAYPFEDQD
OsSAPK5     DGSPAPRLKICDFGYS.......KSSLLHSRPKSTVGTPAYIAPEVLSRREYDGKHADVWSCGVTLYVMLVGAYPFEDPK
OsSAPK6     DGSPAPRLKICDFGYS.......KSSILHSKPKSTVGTPAYIAPEVLSRREYDGKMADVWSCGVTLYVMLVGAYPFEDPD
OsSAPK7     DGSPAPRLKICDFGYS.......KSSILHSKPKSTVGTPAYIAPEVLSRREYDGKTADVWSCGVTLYVMLVGAYPFEDPD
OsSAPK8     DGSPAPRLKICDFGYS.......KSSVLHSQPKSTVGTPAYIAPEVLLKKEYDGKTADVWSCGVTLYVMVVGAYPFEDPE
OsSAPK9     DGSTAPRLKICDFGYS.......KSSVLHSQPKSTVGTPAYIAPEVLLKKEYDGKIADVWSCGVTLYVMLVGAYPFEDPE
OsSAPK10    DGSTAPRLKICDFGYS.......KSSVLHSQPKSTVGTPAYIAPEVLLKKEYDGKIADVWSCGVTLYVMLVGAYPFEDPD
SbSnRK2.1   DGSVAPRLKICDFGYS.......KSSVLHSQPKSTVGTPAYIAPEVLSRKEYDGKVADVWSCGVTLYVMLVGAYPFEDPD
SbSnRK2.2   DGSIAPRLKICDFGYS.......KSSVLHSQPKSTVGTPAYIAPEVLARKEYDGKVADVWSCGVTLYVMLVGAYPFEDPD
SbSnRK2.3   DGSPTPRVKICDFGYS.......KSALLHSKPKSTVGTPAYIAPEVLSRKEYDGKVADVWSCGVTLYVMLVGSYPFEDPE
SbSnRK2.4   DGSPAPRLKICDFGYS.......KSSVLHSRPKSAVGTPAYIAPEVLSRREYDGKLADVWSCGVTLYVMLVGAYPFEDPD
SbSnRK2.5   DGSPAPRLKICDFGYS.......KSSVLHSRPKSTVGTPAYIAPEVLSRREYDGKHADVWSCGVTLYVMLVGAYPFEDPK
SbSnRK2.6   DGSPAPRLKICDFGYS.......KSSILHSKPKSTVGTPAYIAPEVLSRREYDGKMADVWSCGVTLYVMLVGAYPFEDPD
SbSnRK2.7   DGSPAPRLKICDFGYS.......KSSILHSKPKSTVGTPAYIAPEVLSRREYDGKTADVWSCGVTLYVMLVGAYPFEDPD
SbSnRK2.8   DGSDAPRLKICDFGYS.......KSSVLHSQPKSTVGTPAYIAPEVLLKKEYDGKIADVWSCGVTLYVMVVGAYPFEDPE
SbSnRK2.9   DGSTAPRLKICDFGYS.......KSSVLHSQPKSTVGTPAYIAPEILLNKEYDGKIADVWSCGVTLYVMLVGGYPFEDPE
SbSnRK2.10  DGSTAPRLKICDFGYS.......KSSVLHSQPKSTVGTPAYIAPEVLLKKEYDGKIADVWSCGVTLYVMLVGAYPFEDPD
SbSnRK2.11  DGSPAPRLKICDFGYS.......KSSVLHSRPKSAVGTPAYIAPEVLSRREYDGKLADVWSCGVTLFVMLVGAYPFEDQD
SoSnRK2.1   DGSVAPRLKICDFGYS.......KSSVLHSQPKSTVGTPAYIAPEVLSRKEYDGKVADVWSCGVTLYVMLVGAYPFEDPD
SoSnRK2.2   DGSIAPRLKICDFGYS.......KSSLLHSQPKSTVGTPAYIAPEVLARKEYDGKVADVWSCGVTLYVMLVGAYPFEDPD
SoSnRK2.3   DGSPTPRVKICDFGYS.......KSGLLHSKPKSTVGTPAYIAPEVLSRKEYDGKVADVWSCGVTLYVMLVGSYPFEDPE
SoSnRK2.4   DGSPAPRLKICDFGYS.......KSSVLHSRPKSAVGTPAYIAPEVLSRREYDGKLADVWSCGVTLYIMLVGAYPFEDPD
SoSnRK2.5   DGSPAPRLKICDFGYS.......KSSVLHSRPKSTVGTPAYIAPEVLSRREYDGKHADVWSCGVTLYVMLVGAYPFEDPK
SoSnRK2.6   DGSPAPRLKICDFGYS.......KSSILHSKPKSTVGTPAYIAPEVLSRREYDGKMADVWSCGVTLYVMLVGAYPFEDPD
SoSnRK2.7   DGSPAPRLKICDFGYS.......KSSILHSKPKSTVGTPAYIAPEVLSRREYDGKTADVWSCGVTLYVMLVGGYPFEDPD
SoSnRK2.8   DGSDAPRLKICDFGYS.......KSSVLHSQPKSTVGTPAYIAPEVLLKKEYDGKIADVWSCGVTLYVMVVGAYPFEDPE
SoSnRK2.9   DGSTAPRLKICDFGYS.......KSSVLHSQPKSTVGTPAYIAPEVLLNKEYDGKIADVWSCGVTLYVMLVGGYPFEDPE
SoSnRK2.10  DGSTAPRLKICDFGYS.......KSSVLHSQPKSTVGTPAYIAPEVLLKKEYDGKVADVWSCGVTLYVMLVGAYPFEDPD
```

```
               210       220       230       240       250       260       270
AtSnRK2.1   DPKNFRKTIQRIMAVQYKIPDYVHISQECKHLLSRIFVTNSAKRITLKEIKNHPWYLKNLPKELLESAQAAYYKR.....
AtSnRK2.2   EPRDYRKTIQRILSVTYSIPEDLHISPECRHLISRIFVADPATRITIPEITSDKWFLKNLPGDLMDE....NRMGS....
AtSnRK2.3   EPRDYRKTIQRILSVKYSIPDDIRISPECCHLISRIFVADPATRISIPEIKTHSWFLKNLPADLMNE....SNTGS....
AtSnRK2.4   DPKNFRKTIQKIMAVQYKIPDYVHISQDCKNLLSRIFVANSLKRITIAEIKKHSWFLKNLPRELTETAQAAYFKK.....
AtSnRK2.5   DPRNFRKTIQRIMAVQYKIPDYVHISQECRHLLSRIFVTNSAKRITLKEIKKHPWYLKNLPKELTEPAQAAYYKR.....
AtSnRK2.6   EPKNFRKTIHRILNVQYAIPDYVHISPECRHLISRIFVADPAKRISIPEIRNHEWFLKNLPADLMND....NTMTT....
AtSnRK2.7   DPRNIRNTIQRILSVHYTIPDYVRISSECKHLLSRIFVADPDKRITVPEIEKHPWFLKGPLVVPPEEEKCDNGVEE....
AtSnRK2.8   DPKDFRKTIGRILKAQYAIPDYVRVSDECRHLLSRIFVANPEKRITIEEIKNHSWFLKNLPVEM.....YEGSLM.....
AtSnRK2.9   DPRNFRKTVQKIMAVNYKIPGYVHISEDCRKLLSRIFVANPLHRSTLKEIKSHAWFLKNLPRELKEPAQAIYYQR.....
AtSnRK2.10  DPKNFRKTIQKIMAVKYKIPDYVHISQDCKHLLSRIFVTNSNKRITIGDIKKHPWFLKNLPRELTEIAQAAYFRK.....
BdSnRK2.1   EPRNFRKTITRILSVQYSIPDYVRVSTECIHLLSRIFVGNPEQRITIPEIKNHPWFLKNLPVEMTDE..YQRSMQ.....
BdSnRK2.2   EPKNFRKTITRILSVQYAVPDYVRISMECRHLLSRIFVANPEQRITIPEIKNHPWFLKNLPIEMTDE..YQLRLQ.....
BdSnRK2.3   DPRNFRKTISRILGVQYSIPDYIRVSSDCRRLLSQIFTADPSKRITIPEIKKLPWYLKSLPKEIAERDRPNFTEPEP...
BdSnRK2.4   DPKNIRKTIQRIMSVQYNIPDHVHISTECRQLMARIFVNSPGRRITMKEIKSHPWFLRNLPRELTEAMQAMYYRR.....
BdSnRK2.5   DPKNFRKTIARIMSVQYKIPEYVHVSQPCRHLLSRLFVASPHKRITMAEIKAHPWFLKNLPRELKDEAQQAYYNRR.PPT
BdSnRK2.6   DPKNFRKTIGRIVSIQYKIPEYVHISQDCRQLLSKIFVANPAKRITIREIRNHPWFLKNLPRELTETVQEKYYKK.....
BdSnRK2.7   DPKNFRKTIGRIMSIQYKIPEYVHVSQDCKQLLARIFVANPAKRITMREIRSHPWFLKNLPRELTEPAQAMYYKR.....
BdSnRK2.8   EPKNFRKTIQRILSVQYSIPDNVDISPECRHLISRIFVGDPALRITIPEIRNHGWFLKNLPADLMDD....DSMSS....
BdSnRK2.9   DPKNFKMTIQKILGVQYSIPDYVHISLECRDLISRIFVANPATRITMPEIKNHPWVLKNLPVDLMDG....STVSN....
BdSnRK2.10  EPKNFRKTIQRILSVQYSIPDYVHISTECGDLISKIFVGNPATRITIPEIRNHPWFLKNLPADLIDD....STMSN....
OsSAPK1     DPRNFRKTITRILSVQYSIPDYVRVSADCRHLLSRIFVGNPEQRITIPEIKNHPWFLKNLPIEMTDE..YQRSMQ.....
OsSAPK2     EPRNFRKTITRILSVQYMVPDYVRVSMECRHLLSRIFVANPEQRITIPEIKNHPWFLKNLPIEMTDE..YQMSVQ.....
OsSAPK3     DPRNFRKTISRILGVQYSIPDYVRVSSDCRRLLSQIFVADPSKRITIPEIKKHTWFLKNLPKEISEREKADYKDTD....
OsSAPK4     DPKNIRKTIQRIMSVQYKIPDYVHISAECKQLIARIFVNNPLRRITMKEIKSHPWFLKNLPRELTETAQAMYYRR.....
OsSAPK5     DPKNFRKTISRIMSVQYKIPEYVHVSQPCRHLLSRIFVANPYKRISMGEIKSHPWFLKNLPRELKEEAQAVYY.......
OsSAPK6     DPKNFRKTIGRIVSIQYKIPEYVHISQDCRQLLSRIFVANPAKRITIREIRNHPWFMKNLPRELTEAAQAKYYKK.....
OsSAPK7     DPKNFRKTIGRIMSIQYKIPEYVHVSQDCRQLLSRIFVANPAKRITIREIRNHPWFLKNLPRELTEAAQAMYYKK.....
OsSAPK8     EPKNFRKTIQRILNVQYSIPENVDISPECRHLISRIFVGDPSLRITIPEIRSHGWFLKNLPADLMDD....DSMSS....
OsSAPK9     DPKNFRKTIQKILGVQYSIPDYVHISPECRDLITRIFVGNPASRITMPEIKNHPWFMKNIPADLMDD....GMVSN....
OsSAPK10    EPKNFRKTIQRILGVQYSIPDYVHISPECRDLIARIFVANPATRISIPEIRNHPWFLKNLPADLMDD....SKMSS....
SbSnRK2.1   DPKNFRKTITRILSVQYSIPDYVRVSTECRHLLSRIFVGNPEQRITIPEIKNHPWFLKNLPVEMTDE..YQQSMQQ....
SbSnRK2.2   EPKNFRKTLTRILSVQYAVPDFVRVSMECRHLLSRIFVAIPEQRITIPEIKNHPWFLKNLPIEMTDE..YQMNLQ.....
SbSnRK2.3   DPRNFRKTISRILGVQYSIPDYVRVSSECRRLLSQIFVADPSKRITIPEIKHHPWFLKNLPREISEREKANYKDSD....
SbSnRK2.4   DPKNIRKTIQQIMQVQYKIPDYVHISTECQQLLARIFVANPMRRITMKEIKSHPWYLKNLPRELTETAQGMYYRR.....
SbSnRK2.5   DPKNFRKTISRIMSVQYKIPEYVHVSHNCRHLLSRIFVQNPYKRITMSEIKSHPWFLKNLPRELKEEAQAAYYSRRGVGD
SbSnRK2.6   DPKNFRKTIGRIVSIQYQIPEYVHISQDCRQLLARIFVANPAKRITIREIRNHPWFLKNLPRELTEAAQAKYYKK.....
SbSnRK2.7   DPKNFRKTIGRIMSIQYKIPEYVHVSQDCKELLSRIFVANSAKRITIREIRNHPWFLKNLPRELTEPAQAMYYKK.....
SbSnRK2.8   EPKNFRKTIQRILNVQYAIPDNVNISPECRHLISRIFVGDPAMRITIPEIRNHSWFLKNLPADLMDD....DSMSN....
SbSnRK2.9   DPKNFKKTIQKILGVQYAIPDYVCVSPECRDLISRIFVANPADRITMPEIKSHPWFVKNLPADLVGD....GTVS.....
SbSnRK2.10  DPKNFRKTIQRILGVQYSIPDYVHISPECQDLISRIFVANPATRITIPEIRNHPWFLKNLPADLMDD....STMSK....
SbSnRK2.11  DPKNIRKTIQRIAAIQYTIPDNIHISDDCRQLISRIFVSNPLRRITMREIKNHPWFLKNLPRELTEAVQLSYFRR.....
SoSnRK2.1   DPKNFRKTITRILSVQYSIPDYVRVSTECRHLLSRIFVGNPEQRITIPEIKNHPWFLKNLPIEMTDE..YQQSMQ.....
SoSnRK2.2   EPRNFRKTLTRILSVQYAVPDFVRVSMECRHLLSRIFVANPEQRITIPEIKNHPWFLKNLPIEMTDE..YQRNLQ.....
SoSnRK2.3   DPRNFRKTISRILGVQYSIPDYVRVSSDCRRLLSQIFVADPSKRITIPEIKHHPWFLKNLPREISEREKANYKDAD....
SoSnRK2.4   DPKNIRKTIQQIMQVQYKIPDYVHISTECQQLLARIFVANPMRRIIMKEIKSHPWFLKNLPRELTETAQGMYYRR.....
SoSnRK2.5   DPKNFRKTISRIMSVQYKIPDYVHVSQNCRHLLSRIFVANPYKRISMGEIKSHPWFLKNLPR..............
SoSnRK2.6   DPKNFRKTIGRIVSIQYQIPEYVHISQDCRQLLARIFVANPAKRITIREIRNHPWFLKNLPIELTEAAHAKYYKK.....
SoSnRK2.7   DPKNFRKTIGRIMSIQYKIPEYVHVSQDCKELLSRIFVANSAKRITIREIRNHPWFLKNLPRELTEAAQAMYYKK.....
SoSnRK2.8   EPKNFRKKIQRILNVQYAIPDNVNISPECRHLISRIFVGDPAMRITIPEIPNHSWFLKNLPADLMDD....DSMSN....
SoSnRK2.9   DPKNFKKTIQKILGVQYAVPDYVHVSPECRDLISRIFVANPADRITMPEIKSHPWFVKNLPADLVGD....GTVN.....
SoSnRK2.10  EPKNFRKTIQRILGVQYSIPDYVHISPECQDLISRIFVANPATRITIPEIRNHPWFLKNLPADLMDD....STMSK....
```

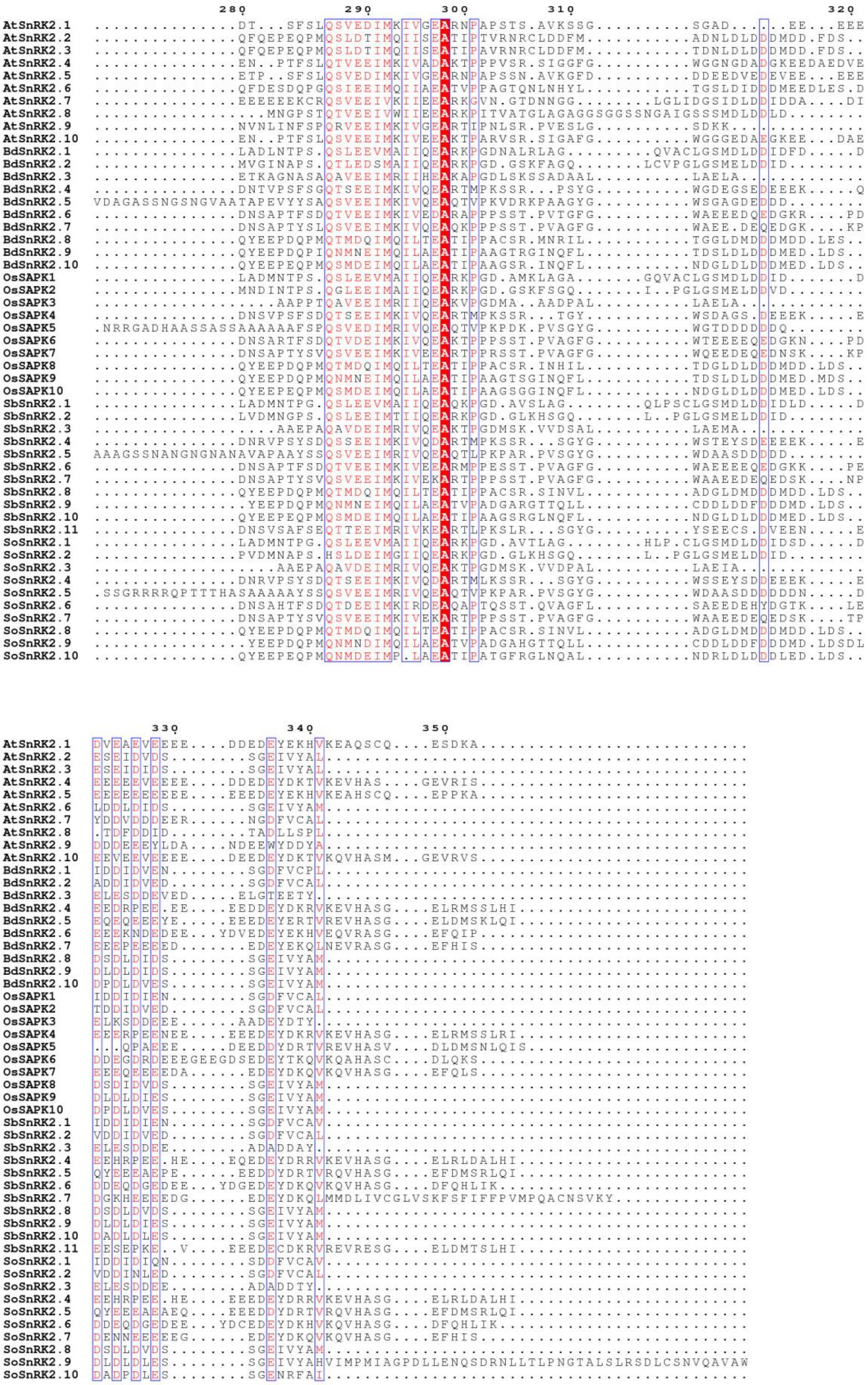

Figure S2 Protein sequence alignment of SoSnRK2s and SnRK2s from other plant species. Numbers on the top indicate amino acid position. Red and yellow background shows sequence identity and sequence similarity in the alignment respectively. Gaps indicated by dashed lines. At, *Arabidopsis thaliana*; Bd, *Brachypodium distachyon*; Os, *Oryza sativa*; Sb, *Sorghum bicolor*; So, *Saccharum officinarum.*
